# Modulation of the Arabidopsis Starch Metabolic Network by the Cytosolic Acetyl-CoA Pathway in the Context of the Diurnal Illumination Cycle

**DOI:** 10.3390/ijms251910850

**Published:** 2024-10-09

**Authors:** Lei Wang, Carol M. Foster, Wieslawa I. Mentzen, Rezwan Tanvir, Yan Meng, Basil J. Nikolau, Eve Syrkin Wurtele, Ling Li

**Affiliations:** 1 College of Life Sciences, Shihezi University, Shihezi 832003, China; wangleibailu@163.com; 2 Department of Biological Sciences, Mississippi State University, Mississippi State, MS 39762, USA; rt916@msstate.edu; 3 Department of Genetics, Development and Cell Biology, Iowa State University, Ames, IA 50011, USA; cmfoster@cox.net (C.M.F.); wimentzen@gmail.com (W.I.M.); 4 Department of Agriculture, Alcorn State University, Lorman, MS 39096, USA; ymeng@alcorn.edu; 5 Roy J. Carver Department of Biochemistry, Biophysics, and Molecular Biology, Iowa State University, Ames, IA 50011, USA; dimmas@iastate.edu; 6 Center for Metabolic Biology, Iowa State University, Ames, IA 50011, USA

**Keywords:** starch, ATP citrate lyase, acetyl-CoA, short day (SD) photoperiod, transcriptomics, starch metabolic network

## Abstract

The starch metabolic network was investigated in relation to other metabolic processes by examining a mutant with altered single-gene expression of ATP citrate lyase (ACL), an enzyme responsible for generating cytosolic acetyl-CoA pool from citrate. Previous research has shown that transgenic antisense plants with reduced ACL activity accumulate abnormally enlarged starch granules. In this study, we explored the underlying molecular mechanisms linking cytosolic acetyl-CoA generation and starch metabolism under short-day photoperiods. We performed transcriptome and quantification of starch accumulation in the leaves of wild-type and antisense seedlings with reduced ACL activity. The antisense-*ACLA* mutant accumulated more starch than the wild type under short-day conditions. Zymogram analyses were conducted to compare the activities of starch-metabolizing enzymes with transcriptomic changes in the seedling. Differential expression between wild-type and antisense-*ACLA* plants was detected in genes implicated in starch and acetyl-CoA metabolism, and cell wall metabolism. These analyses revealed a strong correlation between the transcript levels of genes responsible for starch synthesis and degradation, reflecting coordinated regulation at the transcriptomic level. Furthermore, our data provide novel insights into the regulatory links between cytosolic acetyl-CoA metabolism and starch metabolic pathways.

## 1. Introduction

In plants, storage of carbohydrate, in the form of starch granules, provides a mechanism by which carbon and energy are stored and subsequently remobilized to sustain respiration, metabolism, growth, and development [1,2,3]. The complex regulatory mechanisms that direct the net accumulation of starch, and the integration of this metabolic network with central metabolism are key to the flow of carbon in planta. In leaves, diurnal synthesis and degradation of sugars and starch is thought to mitigate changes in the net pool of carbon metabolites [4]. However, many questions remain about the mechanisms of starch granule production and degradation and its integration with central metabolism [5,6,7,8,9,10,11,12,13,14,15]. Ultimately, clarifying the process of granule biosynthesis and unraveling interactions between starch metabolism and other metabolic pathways may broaden the functionality of starch for human use.

Starch granules accumulate and degrade in leaf chloroplasts during the light and dark cycles, respectively. Only a small amount of starch is retained at the end of a dark period [16,17,18,19,20]. Furthermore, starch accumulation is environmentally regulated, particularly by the length of the photoperiod. Thus, more starch accumulates when plants are grown in a short day (SD) photoperiod than in a long day (LD) photoperiod [17,21,22], providing for more carbon/energy resources during a longer night period [2].

Starch synthesis is initiated by the formation of ADP-glucose, the photosynthetically generated precursor. The accumulation of starch is modulated by the metabolic status of the plant and is sensitive to external and endogenous stimuli. Glucose and sucrose levels [23,24], illumination status [25,26,27], and the circadian clock [28,29] influence regulatory mechanisms that control the expression of genes implicated in balancing the metabolic production and consumption of starch. Turnover of starch in different tissues and organs in response to environmental fluctuations and nutrient availability is integrated by this regulatory network, thus maintaining the carbon balance throughout the plant [30,31,32].

One approach to understanding starch metabolism and its regulation in the context of other metabolic processes is to investigate single-gene mutants that perturb metabolism and characterize how starch accumulation is affected. One such mutation is in gene(s) that affect ATP citrate lyase (ACL) expression. ACL is the hetero-octomeric enzyme, composed of four A subunits and four B subunits (called ACLA and ACLB, respectively) [33,34,35]. ACL is an enzyme component of the citrate shuttle, which produces the cytosolic acetyl-CoA pool from exported mitochondrial citrate [36,37]. The cytosolic acetyl-CoA is the precursor for many biochemical pathways crucial to plant growth and development. Transgenic Arabidopsis antisense-*ACLA* plants that exhibit lower levels of ACL activity exhibit a distinct “bonsai” phenotype [33]. When these plants are grown in LD conditions, the leaves contain abnormally enlarged starch granules [33], indicating an imbalance between starch biosynthesis and mobilization.

To elucidate underlying molecular events involved in starch metabolism and its connection to cytosolic acetyl-CoA metabolism, we analyzed starch accumulation, starch metabolism enzyme activities, and global gene-expression patterns in WT and antisense-*ACLA* plants grown in an SD photoperiod. Our objectives were to determine how decreased cytosolic ACL activity affects the pathways involved in carbon metabolism and may lead to subsequent changes in metabolism throughout the plant.

## 2. Results

### 2.1. Phenotype of Antisense-ACLA Plants Is Dependent on the Length of the Growth Photoperiod

**Figure 1:**
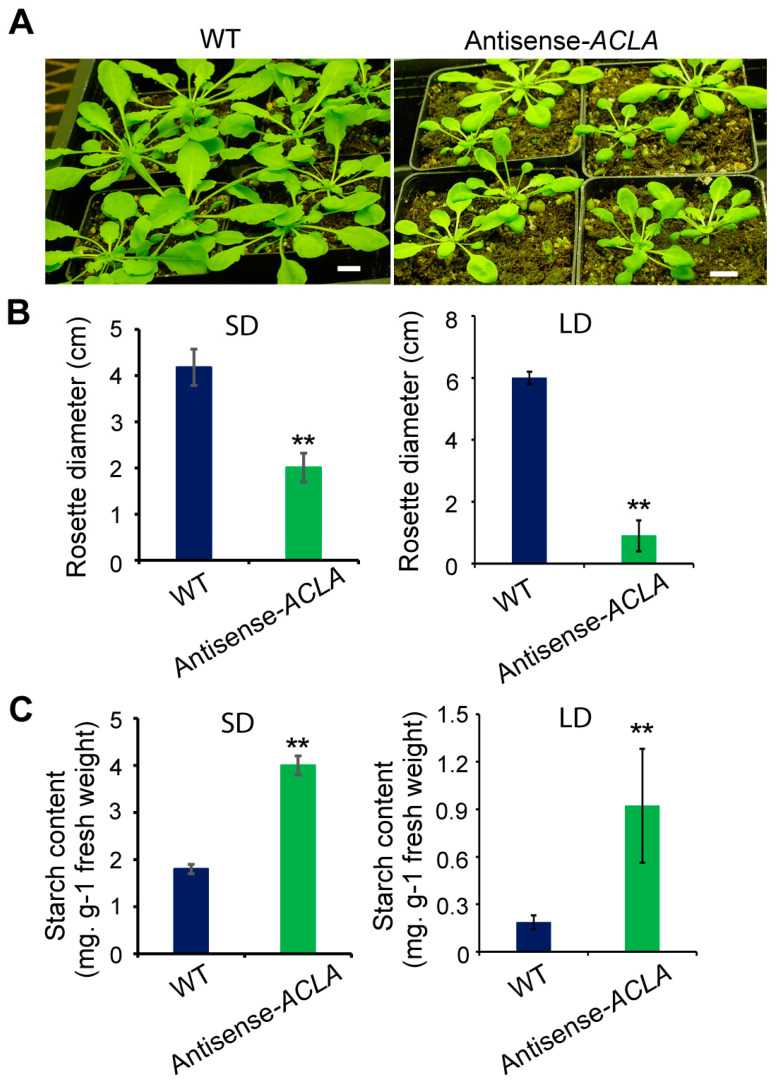
The phenotype of antisense-*ACLA* plants is affected by the length of the light–dark photoperiod. (**A**) Phenotype of antisense-*ACLA* and WT plants grown under SD conditions at 42 days after imbibition. Bar = 1 cm. (**B**) Rosette diameter of antisense-*ACLA* and WT plants grown under SD conditions for 42 days, and LD conditions at 26 days after imbibition. Mean ± S.D., *n* = 16 plants. (**C**) Starch content of antisense-*ACLA* and WT plants grown under SD conditions for 42 days and LD conditions at 26 days after imbibition. Mean ± S.D., *n* = 5 plants, three individual experiments were repeated for analysis. Data under LD conditions in (**B**,**C**) are from a previous publication [33]. Student’s *t*-test was used to compare antisense-*ACLA* and WT plants in (**B**,**C**) (** *p* < 0.01).

The antisense-*ACLA* plants grown under an LD photoperiod present a miniature and bushy “bonsai” phenotype, with very small dark leaves, which accumulate enhanced starch levels [33]. The experiments presented herein indicate that day length affects the penetrance of the antisense-*ACLA* phenotype. Specifically, when antisense-*ACLA* plants were grown under SD conditions, the plants at 42 days after planting (DAP) were smaller than the WT plants (Figure 1A,B). However, the difference between the mutants and WT was less pronounced than under LD conditions. Despite this, starch accumulation in the mutants remained significantly increased, more than doubling that found in WT plants (Figure 1C, *p* < 0.01).

### 2.2. Fluctuation in Transcriptome under SD Diurnal Cycle: The Circadian Rhythm Is Not Altered by ACL Reduction

**Figure 2:**
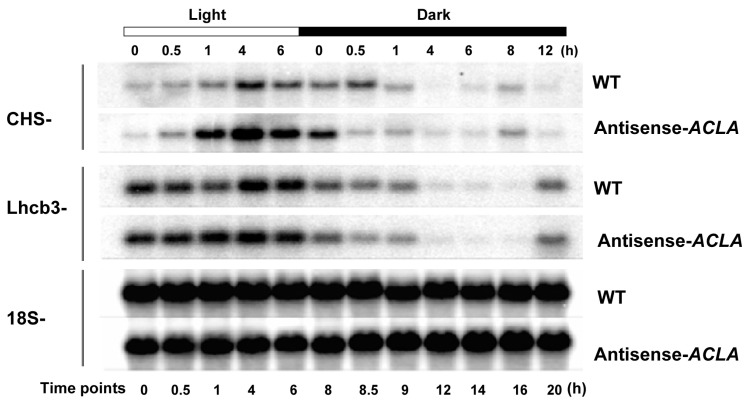
The antisense-*ACLA* mutation does not affect the circadian behavior in the expression of the *Lhcb3* and *CHS* genes. Northern blot analyses of the *Lhcb3*, *CHS*, and 18S RNAs were conducted in antisense-*ACLA* and WT plants at 42 DAI, grown under an SD diurnal cycle. The analysis was repeated in two independent experiments.

We monitored the accumulation of light-harvesting complex (*Lhcb3*) and chalcone synthase (*CHS*) mRNAs in an SD diurnal cycle in both WT and antisense-*ACLA* plants. These two light-regulated genes are known to show a diurnal or circadian rhythm under an LD photoperiod [28], but their expression pattern is unknown in an SD photoperiod. Northern blot analyses showed that both the *Lhcb3* and *CHS* mRNAs display diurnal rhythms under SD, and their circadian expression behavior is unaffected by the antisense-*ACLA* mutation (Figure 2).

**Figure 3:**
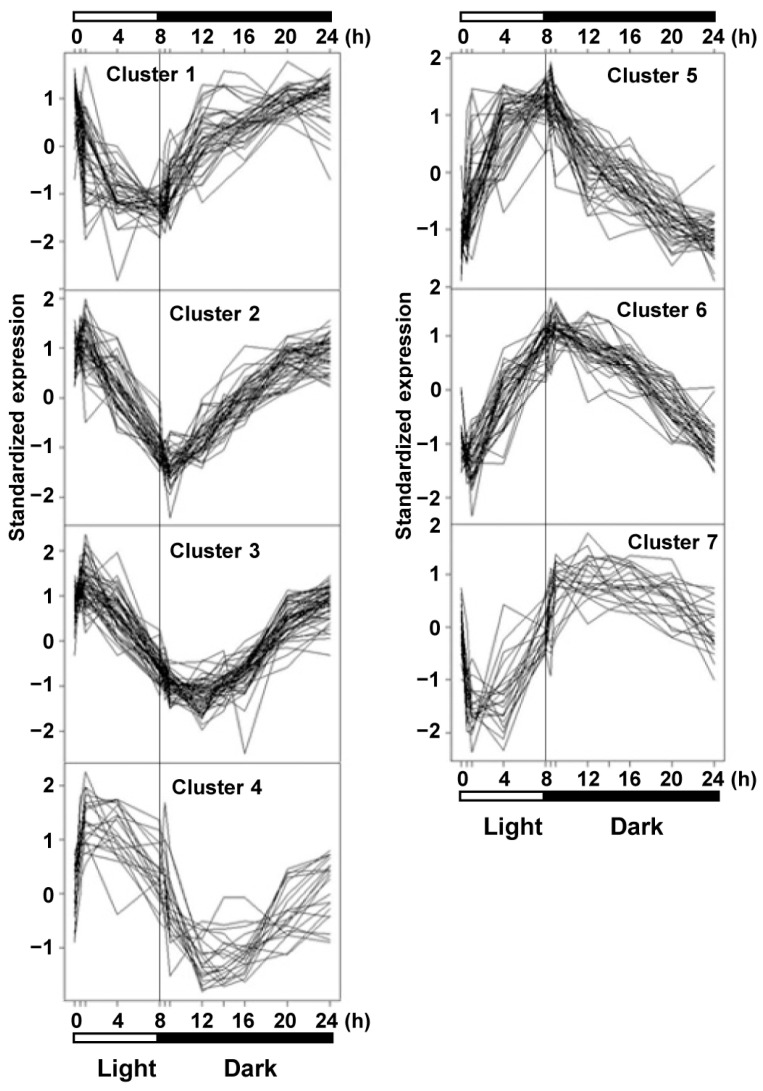
Clustering analysis of 272 differentially expressed genes affected by the circadian rhythm during an SD diurnal cycle in the WT seedlings at 42 DAI. These 272 genes were identified based on the most significant changes in expression during the SD diurnal cycle, with the FDR controlled at 0.00031. The 272 genes were grouped into seven clusters using the K-medoids clustering method [37]. Gene designations are provided in Supplementary Table S1. RNA was analyzed using GeneChip. The analysis was repeated in two independent experiments.

Previous studies reported diurnal changes in the transcriptome of starch metabolic genes in plants grown under an LD photoperiod [35]. Because the penetrance of the antisense-*ACLA* phenotype is reduced when plants are grown in the SD photoperiod, we compared gene expression in the WT plants grown in the SD photoperiod. Using statistical methods [36] that evaluate a *p*-value threshold of 0.00001, and false discovery rate (FDR) controlled at 0.00031, we identified 270 genes that showed significantly differential expressions over the 11 time points that were evaluated in the WT plants. We grouped these genes into seven clusters based on their accumulation patterns, as calculated by the K-medoids clustering method [37] (Figure 3 and Supplementary Table S1).

Clusters with genes showing different diurnal patterns of expression may represent distinct biological processes. This is illustrated, for example, by the genes that occur in Clusters 4 and 7. Specifically, Cluster 4 contains genes with the peak expression occurring in the light period; 25% of these genes code for chloroplast-localized proteins, and none code for mitochondrially localized proteins. In contrast, only ~10% of the Cluster 7 genes, which show their peak expression in the dark period, encode for chloroplast-localized proteins, and ~7.5% of these genes encoding mitochondrial-localized proteins (Supplementary Table S1).

### 2.3. Expression of Genes Coding for Starch Biosynthesis and Degradation Enzymes in an SD Diurnal Cycle

**Figure 4:**
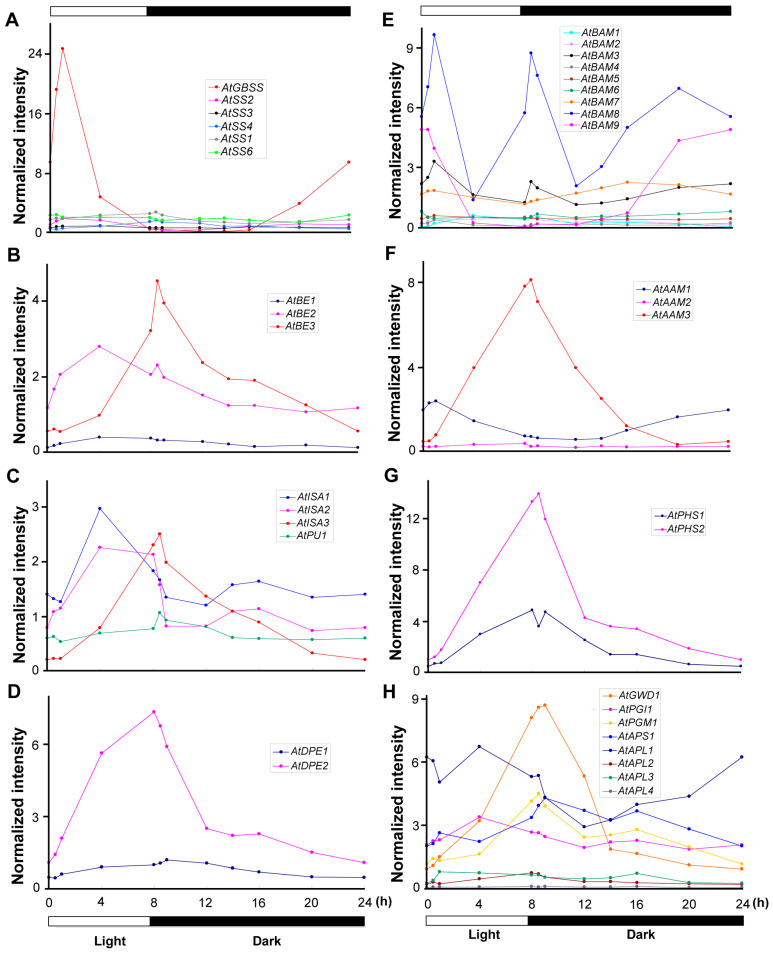
Expression profiles of genes encoding enzymes related to starch metabolism during the SD diurnal cycle. Genes are grouped by their biochemical activity: (**A**) starch synthases (SSs), (**B**) starch branching enzymes (BEs), (**C**) starch debranching enzymes (DBEs), (**D**) starch disproportionating enzymes (DPEs), (**E**) β-amylases (BAMs), (**F**) α-amylases (AAMs), (**G**) starch phosphorylases (PHSs), and (**H**) other enzymes related to starch metabolism. Leaves from WT plants cultivated under short day (SD) conditions, consisting of 8 h of light followed by 16 h of dark, were collected at various times throughout the 24-h cycle. RNA from these samples was then examined using the Affymetrix Arabidopsis ATH1 chip. The average normalized intensity for each microarray chip is 1. RNA was analyzed using GeneChip. The analysis was repeated in two independent experiments.

The expression of genes encoding enzymes involved in starch metabolism fit into three patterns: (1) those that peak in the light period, (2) those that peak in the dark period, and (3) those that peak both in the light and dark periods (Supplementary Figure S1A–C and Table S2). Figure 4 shows the accumulation profiles of mRNAs coding for such starch metabolism enzymes, grouped by their biochemical activity (Supplementary Table S2).

Of particular interest, genes that are in Clusters 5 and 6, which show peak expression at the beginning of the dark period, include multiple genes that are responsive to cold stress (Supplementary Table S1). Specifically, this includes the *DREB1* and *DREB2* gene families, which activate cold-regulated genes or induce drought and/or osmotic stress genes [38] (Supplementary Table S1).

Starch synthases (SSs) transfer a glucosyl unit from ADP-glucose to a growing glucan chain, forming a new α-(1 → 4) linkage [39]. The accumulation patterns of SS mRNAs are distinct from one another (Figure 4A). AtGBSS produces amylose [40], and has a clear induction early in light period. Other SSs are expressed at much lower levels, and there is very little variation over the diurnal cycle. These latter enzymes include those that construct the linear glucan chains of starch (i.e., SS1, SS2, and SS3), as well as the starch initiating enzyme, SS4. Starch branching enzymes (BEs) hydrolyze α-(1 → 4) linkages and introduce α-(1 → 6) linkages into the starch polymer [39]. BE expression correlates with starch accumulation, peaking in the light period (Figure 4B).

Starch debranching enzymes (DBEs) hydrolyze α-(1 → 6) linkages [39]. *AtISA1*, *AtISA2*, and *AtISA3* encode isoamylase-type DBEs, and *AtPU1* encodes a pullulanase-type DBE. Figure 4C shows that *AtISA3* expression peaks at the start of the dark period, which is consistent with its degradative function. *AtISA1* and *AtISA2* show a single mRNA accumulation peak that occurs at the beginning of the dark phase (0.5 h into the dark); this pattern of expression is consistent with their biosynthetic function in the light phase. The *AtPU1* mRNA shows the lowest abundance among the DBEs, and its expression is not affected by the diurnal change in illumination.

Starch disproportionating enzymes (DPEs) catalyze the transfer of glucan residues between linear chains by cleaving and reforming α-(1 → 4) linkages. Thus, in the absence of *DPE* expression, less amylopectin accumulates [39]. *AtDPE1* mRNA accumulation peaks at the onset of the dark phase, and it is expressed at much higher levels than *AtDPE2* (Figure 4D).

Beta-amylases (BAMs) are α-(1 → 4)-specific hydrolases that remove glucan units from the non-reducing end of the starch polymer, stopping at α-(1 → 6) branch linkages. Although primarily involved in starch breakdown [5], elevated transcript levels during both light and dark periods may suggest alternative functions for AtBAM isoforms (Figure 4E). *AtBAM8* has the highest mRNA accumulation of the *BAM* family, with three peaks—early light phase, beginning of the dark phase, and later hours of the dark phase. *AtBAM9*, a plastidial regulator involved in leaf starch degradation [41], peaks at the end of the dark and beginning of the light phase, with the lowest levels at the end of the light phase.

Alpha-amylases (AAMs) are also α-(1 → 4)-specific hydrolases that cleave within linear chains, attacking the starch granule surface to release soluble glucans for further degradation [5]. Of the three AAM isoforms, *AtAAM3* shows the highest mRNA accumulation, which peaks in the transition from the light to dark photoperiod (Figure 4F).

Starch phosphorylases (PHSs) are involved in starch degradation by catalyzing the insertion of a phosphoryl group from inorganic pyrophosphate into an α-(1 → 4) glycosidic bond of the starch polymer, releasing glucose-1-phosphate [5]. *AtPHS2* mRNA accumulation increases throughout the light phase, peaking at the onset of the dark phase, when starch granule disassembly begins (Figure 4G). Subsequently, levels diminish slowly during the dark phase, reaching the lowest level at the end of the dark period. *AtPHS1* mRNA follows a similar pattern, but at lower levels than *AtPHS2*.

Other genes related to starch metabolism include the plastidial glucose-6-phosphate isomerase (PGI1), which catalyzes the interconversion of glucose-6-phosphate and fructose-6-phosphate; glucan water dikinase (GWD1), which catalyzes the addition of phosphate to starch through a dikinase-type reaction mechanism; phosphoglucomutase (PGM1), which catalyzes the interconversion of glucose-1-phosphate and glucose-6-phosphate; and ADP-glucose pyrophosphorylase (ADPG-PP), an allosteric enzyme that catalyzes the synthesis of the starch precursor, ADP-glucose [42,43,44]. Figure 4H shows that most of these enzymes show small diurnal modulations in the SD photoperiod, with the exception of *AtPGI1* and *AtPGM1*, both of which show peak expression at the light-to-dark transition.

### 2.4. Antisense-ACLA Allele Affects Activities of Starch Metabolic Enzymes, without Affecting Their Diurnal Expression Pattern

**Figure 5:**
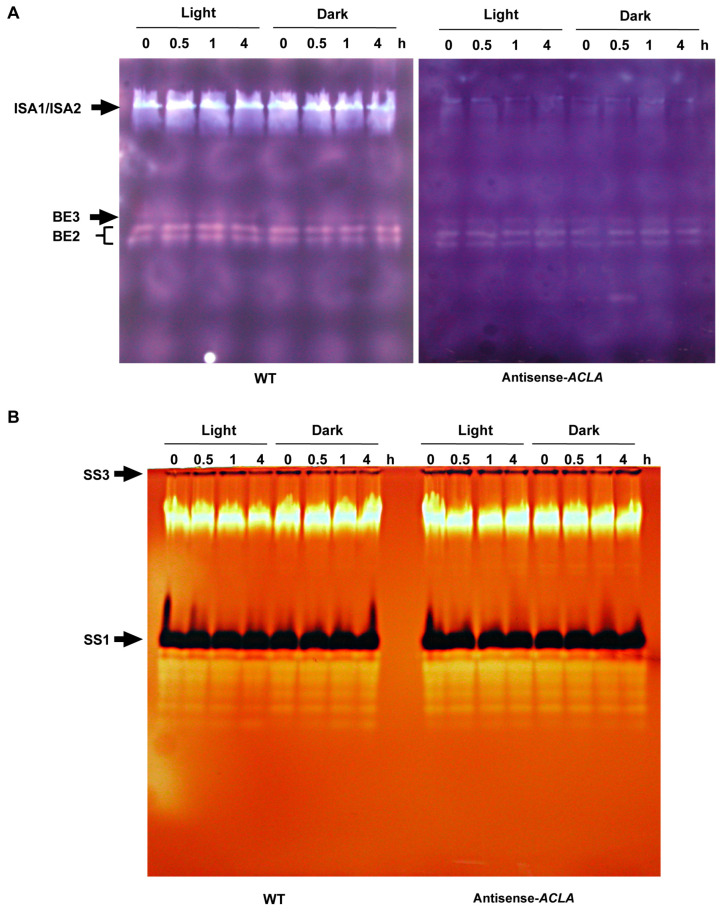
Zymogram analyses of starch modifying and synthesizing activities in extracts from leaves of WT and antisense-*ACLA* plants. (**A**) Protein extracts from 42-DAI leaves were subjected to electrophoresis in a polyacrylamide gel. Proteins were electroblotted to a starch-containing gel. Starch modifying enzyme activities were visualized by staining the gel with I_2_/KI. Activity of DBEs (AtISA1 and AtISA2; light blue bands) and BEs (AtBE3 and AtBE2; red/orange bands). (**B**) Protein extracts were fractionated by electrophoresis in a polyacrylamide gel containing 0.3% glycogen. The gel was incubated with ADP-glucose and stained with I_2_/KI. Dark bands identify the soluble starch synthases (AtSS3 and AtSS1). These experiments were repeated three times, showing very similar results.

The effect of the dominant antisense-*ACLA* allele on starch metabolic enzyme activities was visualized by the use of zymograms [45]. Specifically, protein extracts prepared from the rosette leaves of plants grown under the SD photoperiod were fractionated by electrophoresis in polyacrylamide gels containing solubilized potato starch. By staining the resulting gels with I_2_/KI, the relative migration distances and changes in the color of I_2_/KI-stained starch allowed for the detection and identification of DBEs and BEs [46]. Figure 5A illustrates the identification of the debranching enzymes (AtISA1/AtISA2) and branching enzymes (AtBE1 and AtBE2) [47,48]. The activities of these enzymes appeared unchanged across all time points during the diurnal light–dark cycle. Thus, these enzymes are equally active at night, when net starch degradation occurs, as during the day period, when starch is being synthesized. However, and more significantly, the activities of these starch degradation enzymes are significantly reduced in the antisense-*ACLA* mutant plants.

Analogous experiments conducted with glycogen-containing polyacrylamide gels detected soluble starch synthesis activities [49]. Specifically, such gels were incubated with the starch biosynthesis substrate, ADP-glucose, and stained with I_2_/KI. The dark blue bands in the light brown gel are due to starch, indicating starch synthase activity, with multiple colorless bands representing glycogen-hydrolyzing enzymes. These analyses identified two starch synthase isoforms, AtSS3 and AtSS1 [50,51,52], and several unknown glycogen-hydrolyzing enzymes (Figure 5B). None of these activities was affected by the diurnal light–dark cycle or by the antisense-*ACLA* allele.

### 2.5. The Antisense-ACLA Allele Causes Complex Alterations in Seedling Transcriptome

We profiled the transcriptome of antisense-*ACLA* plants as compared to the WT by microarray. Antisense-*ACLA* mutant and WT plants were grown in parallel under SD conditions and harvested at 42 DAI, and then RNA was extracted at set time points, with two replicates for each time point, in a split-plot experiment design (see Methods section for detail). Due to the diurnal fluctuations in gene expression, we selected three time points for analysis: (1) 1 h after the start of the light period, (2) 0.5 h after the start of the dark period, and (3) 4 h after the start of the dark period. Our comparative analysis focused on genes differentially expressed across all three time points, as well as at individual time points.

**Figure 6:**
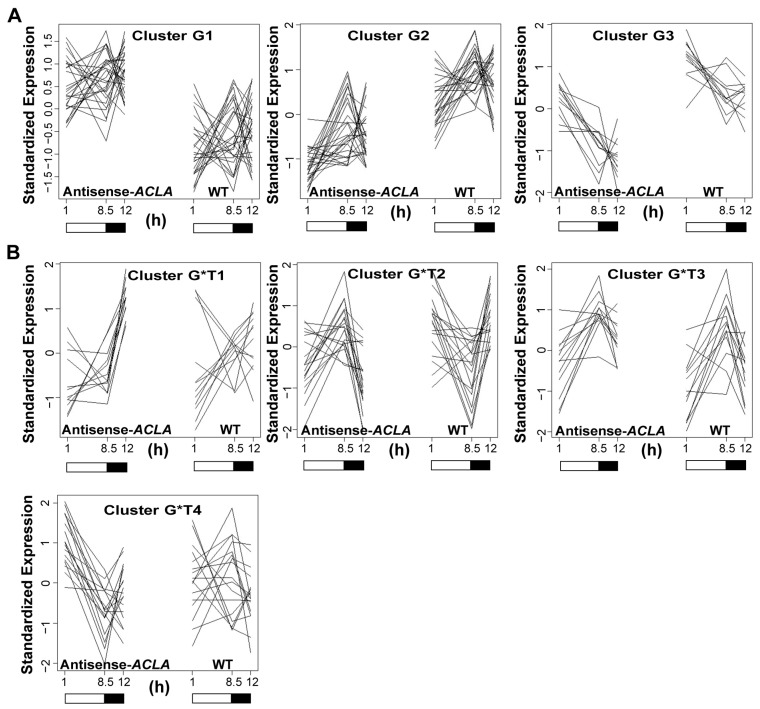
Clustering analysis of gene-expression profiles, with the highest expression differences between WT and antisense-*ACLA* plants grown under an SD diurnal cycle. (**A**) “Genotype” comparison between WT and antisense-*ACLA* plants; gene information is in Supplementary Table S3. (**B**) “Genotype*Time” comparison between WT and antisense-*ACLA* plants; gene information is in Supplementary Table S4. K-medoids clustering method [54] was used to identify the clusters. RNA was analyzed using GeneChip. The analysis was repeated in two independent experiments.

While comparing the global gene-expression profiles across the three time points (i.e., the Genotype comparison), we identified 69 genes that were significantly differentially expressed between the antisense-*ACLA* and WT plants when FDR was controlled at 0.2 [53]. These genes were grouped into three clusters (G1, G2, and G3) using the K-medoids clustering method (Figure 6A and Supplementary Table S3). Although about 15% of the genes within each cluster have unknown functions (i.e., annotated as “expressed protein” or “functions unknown”), we were able to extract biological meanings associated with each cluster, based on the remainder of the genes whose functions had been characterized. Specifically, genes in Cluster G1 showed increased expression in the antisense-*ACLA* plants relative to the WT, and these genes are involved in such processes as transcription regulation, protein post-translational modification, cell wall metabolism, secondary metabolism (e.g., flavonoids), lipid metabolism (e.g., steroids), hormone metabolism (e.g., auxin and ethylene), and stress response. Genes in Clusters G2 and G3 were expressed at lower levels in the antisense-*ACLA* plants than WT, and they are involved in such processes as stress response, transcription regulation, cell wall metabolism, secondary metabolism (e.g., flavonoids), hormone metabolism (e.g., auxin), and protein metabolism.

In the “Genotype*Time” comparison, which examines the interaction between genotype (wild type vs. antisense-*ACLA*) and various time points in the diurnal cycle, 61 genes showed significant differences in expression when FDR was restricted at 0.45 [53]. This analysis helps determine how gene-expression changes over time differ between the two genotypes. The K-medoids clustering method identified four gene clusters, labeled G*T1, G*T2, G*T3, and G*T4 (Figure 6B and Supplementary Table S4). These clusters contain genes that show expression patterns that differ from the “Genotype” comparison. The genes in Clusters G*T1, G*T2, and G*T4 show expression patterns in which the diurnal cycle pattern is flipped by the antisense-*ACLA* mutation, as compared to that shown in the WT plants. These genes are annotated as being involved in protein post-translational modification, cell wall metabolism, hormone metabolism, and stress response. Cluster G*T3 contains genes encoding glycosyl hydrolase family of proteins.

In addition, we used MetNetDB [55] to analyze these transcriptomics data in the context of annotated metabolic pathways. A gene was considered to have altered expression between the antisense-*ACLA* and WT plants if, at any single time point, the error bars in the expression level of that gene did not overlap between the two genotypes. Based on the resulting lists of the up-regulated and down-regulated genes, several metabolic pathways were identified to be affected by the antisense-*ACLA* allele (Table 1). These included pathways of fatty acid elongation, cutin biosynthesis, mevalonate pathway, biotin biosynthesis, brassinosteroid biosynthesis, and chlorophyll biosynthesis, which were up-regulated. And pathways that were down-regulated included brassinosteroids signaling; abscisic acid/Indole-3-acetic acid/jasmonic acid biosynthesis; sulfur-containing amino acid biosynthesis (e.g., homocysteine and cysteine interconversion and cysteine biosynthesis); APX1 (ascorbate peroxidase) signal transduction; pathways that regulate active oxygen levels, including ascorbate glutathione pathway (cytosol, mitochondria, and plastid stroma); ascorbate biosynthesis; and the ascorbate glutathione cycle.

**Figure 7:**
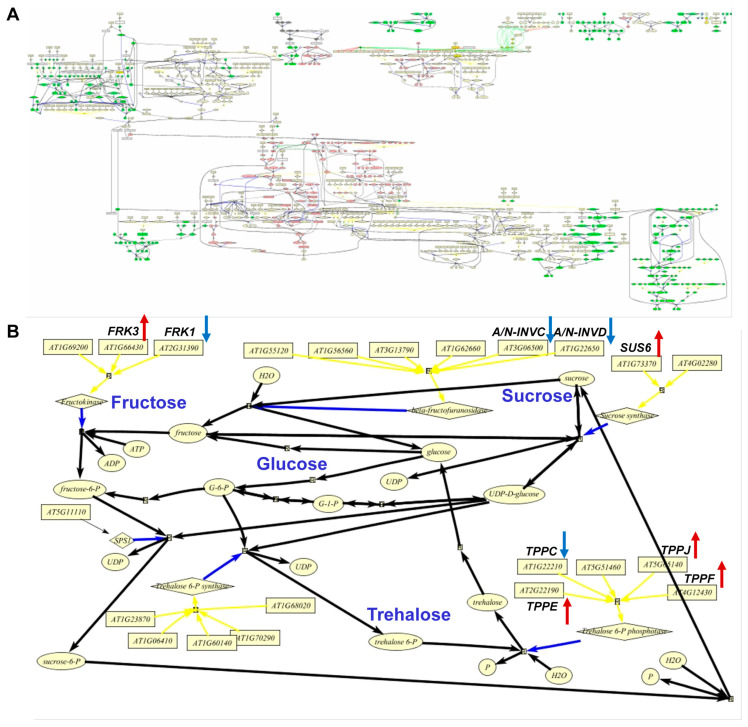
Metabolic networks from FCModeler. (**A**) Metabolic network of carbohydrate and cell wall metabolism. (**B**) Selected pathway to show genes with altered RNA accumulation in sucrose and trehalose metabolism. Red arrows indicate genes that are up-regulated, and blue arrows indicate genes that are down-regulated in antisense-*ACLA* plants grown under an SD diurnal cycle. RNA was analyzed using GeneChip. The analysis was repeated in two independent experiments.

Moreover, we focused on metabolic pathways that direct carbon to sugars and starch accumulation and on pathways that are sensitive to carbon signaling. These analyses identified 35 genes that are involved in trehalose and cell wall metabolism, which showed altered expression over the three time points that were evaluated (Table 2). These genes exhibited at least a 2-fold change in expression between antisense-*ACLA* and WT at one or more time points. Collectively, therefore, these differentially expressed genes identify processes involved in cell wall, trehalose, starch, sucrose, and acetyl-CoA metabolism pathways, which can be integrated into a small network (Figure 7A), as illustrated by the data shown in Figure 7B.

### 2.6. Co-Expression of Starch Metabolic Genes across Diverse Microarray Experiments

We also compared expression profiles of starch metabolism genes from a co-normalized set of publicly available microarray data available at the Nottingham Arabidopsis Stock Centre microarray (NASCArray) database [56,57]. These data were collected from nearly 1000 chips representing 70 Arabidopsis Affymetrix ATH1 microarray experiments. These experiments assessed the expression pattern of ~23,000 Arabidopsis genes, whose expression was modified by numerous perturbations, including mutations, organ types, stage of development, and in response to biotic and abiotic stresses. To facilitate the custom analysis of such a large number of microarray chip data and the associated metadata of each experiment, we used an open-source software, MetaOmGraph (MOG, accessed on 2 January 2020) [58].

**Figure 8:**
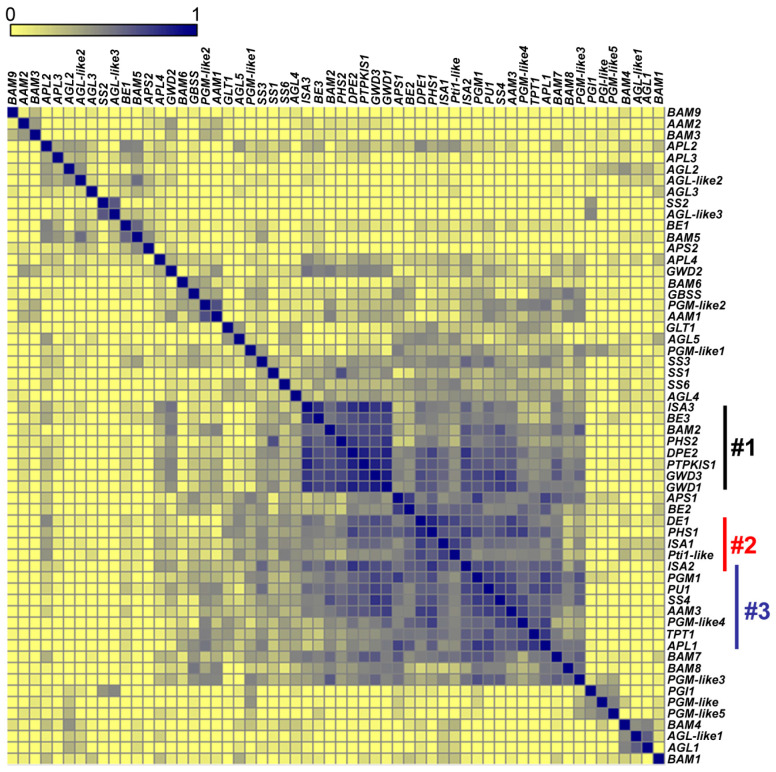
Expression correlations among genes within the starch metabolic network. Expression correlations among starch metabolic genes were evaluated by 963 Affymetrix ATH1 microarray chips representing 70 different Arabidopsis experiments and analyzed by MOG [58]. Pearson correlation values are color-coded. Vertical bars (labeled #1, group 1; #2, group 2; and #3, group 3) denote the three groups of genes with the highest correlations. Group 1 represents genes involved in starch degradation, Group 2 includes genes primarily associated with starch synthesis, and Group 3 comprises genes involved in both starch synthesis and carbohydrate metabolism. Details for each group are described in the text.

Initially, using Pearson correlation as a similarity measure, we compared the expression patterns of 58 genes that are known to be members of the starch metabolic network, which would be expected to show high correlations. These included starch metabolic genes and putative regulators of starch metabolism (i.e., *PTPKIS1* and Pti-like protein). The majority of the selected genes (53 out of the 58 genes) showed the expected high correlation (Figure 8). The five genes that showed low correlations with the other members of the starch metabolic network included (*BAM9*, *AAM2*, *BAM3*, *BAM6*, and *BAM1*).

These correlation calculations identified three distinct groups of strongly co-expressing genes (Figure 8):

**Group 1**: *DPE2*, *GWD1*, *GWD3*, *PHS2*, *SEX4*, *ISA3*, *BAM2*, and *BE3*.
**Group 2**: The starch synthesis genes (*ISA1* and *ISA2*), *DPE1*, *PHS1*, and the gene encoding Pti1-like protein.
**Group 3**: *ISA2*, *SS4*, and *APL1* (At5g19220) involved in starch synthesis; *PU1* and *AAM3* (chloroplastic, glycogen catabolism) linked to degradation; and triose phosphate/phosphate translocator gene (*TPT1*, At5g46110), phosphoglucomutase 1 (*PGM1*, At5g51820), and a PGM-like4 (At1g70820) critical to carbon flux.

Genes in Group 1 are primarily involved in starch degradation, whereas Group 2 genes are associated with starch synthesis and its regulation, and Group 3 genes are a mixture of both starch synthesis and degradation genes, as well as genes associated with broader carbohydrate metabolic processes. Collectively, these genes represent distinct yet interconnected roles in the metabolic processes governing starch synthesis, degradation, and overall carbohydrate metabolism in plants.

Similarly, using Pearson correlation as a similarity measure, we searched for genes with expression patterns similar to *ISA1* (At2g39930). We chose the *ISA1* gene for these analyses because it plays an important role in starch biosynthesis, possibly as a member of a protein complex with *ISA2* (At1g03310) [59]. In addition, in plants grown in SD conditions, the accumulation for both *ISA1* and *ISA2* mRNA peaked during the light phase (Figure 4C). These analyses enabled us to identify potential starch synthesis genes by the correlation with *ISA1* expression, and this would include the expected high correlation with the *ISA2* mRNA. 

Indeed, the expression of *ISA2* showed the highest correlations with the *ISA1* mRNA (Table 3). In addition, eight genes showed a correlation coefficient with *ISA1* mRNA that was higher than 0.66 (Table 3). Notably, three of these genes, *DPE1* (At5g64860), *PHS1* (At3g29320), and ROOT CAP 1 (*RCP1*, At5g17520), are known to participate in starch degradation [60,61]. *RCP1*, previously known as *MEX1*, is a chloroplastic maltose transporter that is critical during starch degradation. *Pti1* (At1g26150) encodes a putative serine/threonine kinase, similar to Pto kinase interactor 1 [62], which is involved in plant–pathogen interactions [62,63]. The high correlation between the *Pti1* kinase and *ISA1* suggests that phosphorylation is important during starch metabolism. Other genes that co-express with *ISA1* include D-cysteine desulfhydrase (At1g48420), which breaks down cysteine to produce pyruvate, ammonia, and sulfuric acid in mitochondrion [64], and two genes with unknown functions, mitochondrial At3g60810 and chloroplastic At4g10470.

**Figure 9:**
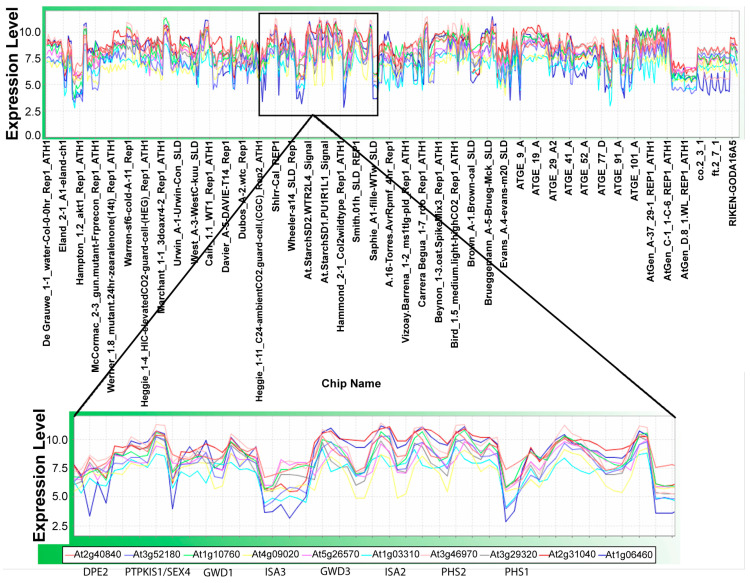
Gene expression correlated with the expression of the *DPE2* gene. Gene-expression profiles of nine genes (*PTPKIS1*/*SEX4*, *GWD1*, *ISA3*, *GWD3*, *ISA2*, *PHS2*, *PHS1*, AT1G06460, and AT2G31040) correlated to the expression profile of *DPE2* (Pearson’s correlation coefficients of between 0.72 and 0.84). Gene expression was evaluated at the level of RNA accumulation from 963 Affymetrix ATH1 microarray chips representing 70 different Arabidopsis experiments and analyzed by MOG [58]; the average expression level for each chip is 6.6.

An additional expression correlation study was conducted with the *DPE2* gene (Figure 9). We selected the *DPE2* gene because the *DPE2* mRNA accumulates during the dark phase of growth [65,66]. Using MOG-based analysis, we found expression profiles for nine genes (correlation coefficients from 0.72 to 0.84) to be similar to *DPE2* (Figure 9). Six of these genes—*GWD1* (At1g10760), *GWD3* (At5g26570), *ISA2* (At1g03310), *ISA3* (At4g09020), *PHS1* (At3g29320), and *PHS2* (At3g46970)—are also involved in starch degradation. *DPE2* showed the highest correlation with *PTPKIS1*/*SEX4*, At3g52180, a novel tyrosine phosphatase that dephosphorylates proteins associated with the SnRK complex [67]. Inactivation of the SnRK complex affects vital metabolic enzymes, such as sucrose synthase and sucrose phosphate synthase, and may alter global responses to carbon and stress signaling [68]. Our data align with previous findings [69] from the Stanford Microarray Facility, showing that *PTPKIS1* and *PHS2* have similar expression profiles. The strong correlation of *PTPKIS1* expression with the *DPE2* co-expression group suggests a link between *PTPKIS1* and carbohydrate metabolism, a hypothesis confirmed by experimental data [70]. *SEX4* is involved in starch degradation.

## 3. Discussion

At the biochemical level, a metabolic process can be defined as a network of enzymes and their regulators that collectively results in the net conversion or accumulation of defined metabolites. Additionally, the advent of whole-genome expression profiling technologies has provided a means of defining biological processes as a collection of co-expressing genes [71,72,73,74]. In this paper, we considered these two definitions in the context of the starch metabolic network. Starch accumulation is the balance between anabolic processes that assemble the polymer from ADP-glucose and catabolic processes that depolymerize starch to generate sugar monomers. In photosynthetic leaf tissue, the balance between anabolic and catabolic divisions of the starch network is diurnally regulated [75,76], resulting in the net accumulation of starch during the light period, which is degraded during the dark period in order to maintain cellular respiration, metabolism, growth, and development. In this context, starch anabolism requires the following gene-products: ADP-glucose pyrophosphorylase (AGPase), SS, BE, and DBE. And starch catabolism requires the following gene products: AAM, BAM, DBE, DPE, and PHS.

In this study, we sought to expand the starch metabolic network by applying global transcriptomic analysis to a genetic mutant (i.e., the antisense-*ACLA* mutant) and environmental modifiers (i.e., SD diurnal photoperiod), which perturb the normal patterns of starch accumulation. Specifically, we followed up on the discovery that reducing the supply of the cytosolic acetyl-CoA-pool by the transgenic expression of a dominant antisense-*ACLA* allele results in the hyperaccumulation of leaf starch [33]. Hence, because the cytosolic acetyl-CoA metabolic network is distinct from the starch metabolic network, we hypothesized that genes whose expressions are altered by the antisense-*ACLA* allele and whose expressions are correlated with the known members of the starch metabolic network could be considered as members of an expanded starch metabolic network. However, the transgenic antisense-*ACLA* allele affects not only starch accumulation but also a range of other morphological traits, which could complicate the analysis. In this study, we found that when the antisense-*ACLA* plants were grown in an SD photoperiod, their abnormal morphological phenotypes were nearly eliminated, yet the hyperaccumulation of leaf starch was still maintained.

It is well known that there are major metabolic changes that occur in the light–dark transition during a diurnal cycle, particularly associated with photosynthetically derived central carbon metabolism, including the accumulation of starch [76], amino acids [77], and fatty acids [78]. Indeed, a prior transcriptomic analysis performed during an LD diurnal cycle identified that both transcriptional and post-translational regulatory mechanisms modulate the expression of the starch metabolic network genes and thereby affect the cyclical daily pattern of starch accumulation in leaves [38]. In the study presented herein, with plants grown in an SD diurnal cycle, the genes that showed the most modulation during the diurnal cycle were grouped into seven co-expressing gene clusters. Many of these clusters appear to be enriched with genes that are associated with stress responses. For example, the co-expressing genes *COR*, *ERD*, *CCR*, and *AtGRP* in Clusters 5 and 6 are known to be cold-regulated [79,80,81].

In contrast to the seven patterns of co-expression obtained with the genes that show the most modulation during the diurnal cycle, the genes of the starch metabolic network showed three patterns of co-expression. Two of these patterns are consistent with the diurnal cycle of starch accumulation and degradation. Namely, genes of starch degradation (i.e., *DPE2*, *GWD1*, *GWD3*, *PHS2*, *SEX4*, *ISA3*, *BAM2*, and *BE3*) are co-expressed and showed peak expression in the dark period, whereas the genes involved in starch biosynthesis (i.e., *ISA1*, *ISA2*, *PHS1,* and *DPE1*) are co-expressed and showed peak expression in the light period. The zymogram analysis of starch anabolic and catabolic enzymes does not necessarily reflect the diurnal changes observed for the transcript of these genes, suggesting that a post-transcriptional mechanism regulates the processes of starch biosynthesis and catabolism. Moreover, these zymograms indicate that the increased accumulation of starch in the antisense-*ACLA* plants is due to diminished starch degradation activity, while starch synthase activity remains unchanged.

The antisense-*ACLA* allele caused changes in the expression of genes involved in pathways that rely on acetyl-CoA as a substrate. These include the up-regulation of several genes involved in fatty acid elongation, secondary metabolite production (e.g., cutin), and hormone biosynthesis (e.g., brassinosteroids). However, the antisense-ACLA allele also generated the down-regulation of genes involved in stress-induced hormone pathways (e.g., jasmonic acid and abscisic acid synthesis and cytokinin degradation), suggesting a complex interplay between acetyl-CoA availability and stress response pathways.

Several changes in gene expression in the antisense-*ACLA* plants suggest additional modulators of stress pathways. These include the down-regulation of the *APX1* and *PMSR2* (peptide methionine sulfoxide reductase 2) genes, which are involved in the oxidative stress response [82,83,84,85], and the up-regulation of the *TPP* gene that is involved in trehalose metabolism, a known stress indicator [86,87,88]. These indicators of altered redox-state stress may be a mechanism of the redox-mediated activation of AGPase, leading to increased starch accumulation. Additionally, the reduced expression of ACL activity induced changes in cell wall metabolism, indicated by the altered expression of genes involved in cell wall synthesis, modification, and degradation. Thus, the reduced supply of cytosolic acetyl-CoA may not only affect starch metabolism but other critical cellular processes.

In summary, the reduced expression of ACL caused by the antisense-*ACLA* allele leads to a complex cascade of metabolic and regulatory changes, resulting in increased starch accumulation, altered stress responses, and modified cell wall metabolism. These findings underscore the intricate interconnections between cytosolic acetyl-CoA metabolism, starch synthesis and degradation, and plant stress responses, offering new insights into the regulatory networks that govern plant metabolism.

## 4. Materials and Methods

### 4.1. Plant Materials, Growth Conditions, and Experimental Design

All *Arabidopsis thaliana* plants used in this study were of the ecotype Columbia (Lehle Seeds, Round Rock, TX, USA). Transgenic plants expressing the antisense *ACLA* sequence, which is using the constitutive CaMV 35S promoter, have previously been described [36]. Seeds were surface-sterilized in a 50% (*v*/*v*) bleach and 0.02% (*v*/*v*) Triton X-100 solution for 7 min and were rinsed three times with sterile distilled H_2_O. Antisense-*ACLA* and wild-type seeds were germinated on a solid growth medium in (100 mm × 15 mm) Petri dishes, with and without 30 mg/mL kanamycin, respectively. The growth medium was buffered with 2.56 mM MES at pH 5.7 and contained 4.3 g/L Murashige and Skoog salts (GibcoBRL, Life Technologies, Rockville, MD, USA), 1% (*w*/*v*) sucrose (Sigma, St. Louis, MO, USA), 1X B5 vitamins (100 mg/mL myo-inositol, 1 mg/mL pyridoxine hydrochloride, 1 mg/mL nicotinic acid, 10 mg/mL thiamine hydrochloride), and 0.6% (*w*/*v*) agar gel (Sigma, St. Louis, MO, USA). The Petri dishes were placed in a growth chamber (73% RH at 22 ± 1 °C) (Model LT105; Percival Scientific, Perry, IA, USA) under a short day (SD) photoperiod (8 h light /16 h dark), with illumination provided by fluorescent lamps (129 ± 16 µM·m^−2^·s^−1^ PAR). After 21 d, seedlings were transplanted to a sterile potting medium (LC1 Sunshine Mix, Sun Gro, Horticulture, Inc., Bellevue, WA, USA) and fertilized with 1X Nutriculture Grower’s Special Blend 21-8-18^PLUS^ (Plant Marvel Labs, Chicago, IL, USA). The antisense-*ACLA* seedlings exhibiting features characteristic of the antisense “bonsai” phenotype [36] were transplanted to 3-inch square pots, two seedlings per pot.

Pots were arranged in 16 randomized blocks (flats), using PROC PLAN of SAS (SAS Institute, Cary, NC, USA), and returned to the same growth chamber and environmental conditions that were used for germinating the seeds. Each flat consisted of plants of the same genotype (8 flats of WT and 8 flats of antisense-*ACL*), and with 21 pots in one flat. At 6 weeks after germination (prior to flower bolting), rosette leaves (#5 to #8 according to Bowman (1994)) that are mature and photosynthetically active [89,90] were harvested from 2 plants from one pot from each flat. These tissue harvests occurred at 0, 0.5, 1, 4, 6, 8, 8.5, 9, 12, 14, 16, and 20 h during the SD photoperiod; the zero time point was at the start of the photoperiod. Thus, 0, 0.5, 1, 4, and 6 h time points were collected during the light cycle, and the 8, 8.5, 9, 12, 14, 16, and 20 h time points were collected during the dark cycle. During these latter harvests, plants were illuminated with a faint green light. Samples were frozen in liquid N_2_ immediately after harvest and stored at −80 °C for RNA and protein extraction. For each time point, two samples were harvested of each genotype, and each sample consisted of leaves from sixteen plants. The entire process was completed on two separate occasions to obtain two replications for all genotype and time combinations. This can be viewed as a split-plot experimental design, with genotype as the whole-plot factor and time as the split-plot factor.

### 4.2. Starch Quantification

Plants grown in parallel and in the same conditions as described above were used to generate tissue for starch analysis. At 6 h into the light phase of the photoperiod, just prior to the transition into the dark phase, when starch content is at a maximum, rosette leaf samples, weighing between 250 and 500 mg fresh weight, were harvested from randomly selected plants of each genotype. Three samples, consisting of leaves from five plants, were collected per genotype. Harvested samples were immediately frozen in liquid N_2_ and stored at −80 °C. Starch content of leaf samples was determined as previously described [18], with slight modification as follows. Leaf samples were immersed in 35 mL 80% (*v*/*v*) ethanol and incubated in a boiling water bath for 30 min or until completely de-pigmented. After removal of the solvent, the leaf samples were homogenized in a Kontes tissue grinder and extracted with a second aliquot of 35 mL 80% (*v*/*v*) ethanol. After homogenizing the insoluble material a second time, it was resuspended in water and incubated in a boiling water bath for 30 min. The suspended mixture was digested with amyloglucosidase, and the released α-glucan polymer (soluble and insoluble) was quantified with a commercial assay kit (catalog no. E0207748, R-BioPharm, Darmstadt, Germany). The statistical significance of differences among samples were evaluated with Student’s *t*-test by using JMP 18 software (SAS Institute).

### 4.3. RNA Blot Analysis

RNA was extracted from frozen leaf samples using GibcoBRL TRIzol reagent (Life Technologies, Rockville, MD, USA) according to the protocol provided by the manufacturer, as previously described [91]. RNA concentrations were determined using a Genesys spectrophotometer (Spectronic Instruments, Rochester, NY, USA). RNA samples (10 mg) were denatured for 15 min at 65 °C and subjected to RNA blot analysis, as previously described [92]. The blots were hybridized with a ^32^P-labeled denatured DNA probe. DNA probes of *Lhcb3*, *CHS,* and 18S RNAs were labeled with [^32^P]dCTP by random priming, using GibcoBRL reagents and protocol (Life Technologies, Rockville, MD, USA). The analysis was performed in triplicate to ensure reproducibility. Washed membranes were exposed to a phosphor screen (Molecular Dynamics, Sunnyvale, CA, USA) for 4 d, scanned with a Storm 840 PhosphorImager (Amersham Biosciences, Piscataway, NJ, USA), and visualized with ImageQuant TL 10.1 software (Global Life Sciences Solutions USA LLC, Wilmington, DE, USA).

### 4.4. In Gel Enzyme Activity Analyses of Starch Metabolic Enzymes

Zymogram analyses of starch metabolic enzymes were conducted as previously described [49], with a few modifications. Specifically, to determine starch synthase activity, leaf samples were homogenized with a buffer consisting of 50 mM Tris acetate (Thermo Fisher Scientific, Waltham, MA, USA), pH 7.5, and 10 mM DTT (Thermo Fisher Scientific, Waltham, MA, USA). Extracts, consisting of 200 mg protein, were subjected to electrophoresis in 7% polyacrylamide gels containing 0.3% (*w*/*v*) glycogen, using a Protean II cell (Bio-Rad Laboratories, Hercules, CA, USA). Following electrophoresis for 4.5 h at 4 °C, with a current of 75 mA, starch synthase activity was visualized by incubating the glycogen-containing gel at room temperature for 36 h in a buffer consisting of 100 mM Bicine (pH 8.0), 0.5 M citrate, 25 mM potassium acetate, 0.5 mg/mL BSA, 5 mM ADP-glucose, 5 mM 2-mercaptoethanol, and 20 mg/mL glycogen. After incubation, the gel was stained with 0.2% iodine and 2% potassium iodide (Thermo Fisher Scientific, Waltham, MA, USA) in 10 mM HCl to detect enzymatic activity. In parallel, starch-modifying enzyme activities were visualized by electroblotting proteins onto a polyacrylamide gel containing 0.3% (*w*/*v*) starch, followed by iodine staining to detect the enzymatic activity. Stained gels were scanned immediately with a GS-800 Calibrated Densiometer (Bio-Rad Laboratories, Hercules, CA, USA) and visualized with MagicScan 4.71 software (UMAX Technologies, Dallas, TX, USA). These experiments were repeated three times.

### 4.5. GeneChip Analysis of SD Experiments

RNA was extracted from leaves as described for Northern blot analysis. Northern blot analysis showed that expression patterns were well characterized by 11 of the 12 time points because the 6 h in light time point consistently fell near a line segment connecting the adjacent points. To identify the expression of the genes that might have different temporal patterns over the SD diurnal cycle (especially the starch metabolic genes), the transcript abundance was monitored at 11 time points (time point 6 h in the light was not selected based on the Northern blots analysis), with two biological replicates. Synthesis of labeled cRNAs, hybridization with Affymetrix Arabidopsis ATH1 arrays (22,810 Arabidopsis probe sets, Affymetrix, Santa Clara, CA, USA), and scanning of the probe arrays were performed at the Iowa State University Gene Chip Facility (Ames, IA, USA). Hybridization intensities were collected by an Agilent GeneArray Scanner (Agilent Technologies, Palo Alto, CA, USA). For data analysis, relative expression intensities were generated and normalized with Microarray Suite (MAS) 5.0 software (Affymetrix) in the form of signal values. The signal value, a robust weighted mean of probe fluorescence for a probe set (corrected for nonspecific signal by subtracting mismatch probe values), was calculated using the One-step Tukey’s Biweight Estimate. Global scaling (Affymetrix) was used to normalize the data by adjusting the mean expression level of each chip to a value of 500. Scatter plot analyses of the two replicates were linear, indicating similarity to each other; we thus used the mean value of expression (normalized intensity). FCModeler (Iowa State University, Ames, IA, USA) was used to model and visualize metabolic networks.

### 4.6. Clustering

The data from each Affymetrix Arabidopsis ATH1 chip were normalized by log (signal + 1) transformation. For each gene, we fitted a mixed linear model with fixed effects for time and random effects for replicate to identify genes that exhibited a significant change in expression across 11 time points for WT. We also fitted another mixed linear model with fixed effects for time and genotype, and random effects for replicate to identify genes that exhibited significant changes in expression over the 3 time points per genotype and 2 genotypes surveyed, respectively. When testing for differences over time, we used the more conservative method previously reported [93] to estimate the false discovery rate (FDR) because a large number of genes exhibited significant differences over time. We used the method previously reported [53] to estimate FDR to gain more power for detecting differences in expression when comparing the WT and mutant genotypes. Thresholds for significance based on *q*-values were selected to maintain false discovery below desired rates. After the matrix of estimated expression means was obtained, the matrix of means was standardized so that Euclidean distance would be related to 1-correlation distance. K-medoids clustering was conducted in R (https://www.r-project.org/, accessed 2 January 2020) with the cluster number that Gap analysis recommended. The Gap statistic method [94] calculates the number of clusters by comparing the degree of clustering of the data to that of a simulated uniform (cluster-free) reference distribution.

### 4.7. Correlation Analysis

Arabidopsis Affymetrix ATH1 data from 963 chips were obtained from Nottingham Arabidopsis Stock Centre microarray database [56,57]. The data represent a large number of experiments that studied the effect of development, stress, and different mutations [56,57] using MAS 5.0 (Affymetrix); the data from all the chips were individually scaled to a common mean = 100, excluding top and bottom 2% signal intensities. We averaged the expression values on chips coming from biological replicates after assessing their replicability by comparing the interquartile range of each data set. Pair-wise Pearson correlation coefficients were computed for 58 genes of the starch metabolism pathway across all probes on the Arabidopsis Affymetrix ATH1 chips by using our custom software, MetaOmGraph [58]. From among the 22 K genes represented on the chips, genes correlated above a specified cut-off to a gene of interest were identified and visualized with MOG [58]. Correlation data, generated in R software (http://www.r-project.org/index.html) [95], for starch metabolism genes were clustered by columns and rows in CIMMaker and visualized in Matrix2png [96].

### 4.8. Statistical Analysis

Qualitative and quantitative composition data were collected from a minimum of three biological determinations for each independent transgenic line and each control. Data are presented as mean ± S.D. (Standard Deviation). Statistical significance relative to the control was calculated with Student’s *t*-test or analysis of variance (ANOVA), ** *p* < 0.01.

## 5. Conclusions

Our study demonstrates that reduced cytosolic ACL activity in antisense-*ACLA* plants leads to significant changes in starch metabolism and related biochemical pathways. The observed increase in starch content suggests an imbalance between starch biosynthesis and degradation, affected by up-regulated genes involved in starch synthesis and down-regulated genes involved in starch degradation. The transcriptional analysis revealed that several pathways, including those for fatty acid elongation, secondary metabolite synthesis, and hormone biosynthesis, are also affected, indicating a broader impact on plant metabolism. The altered expression of genes in the APX1 signal transduction pathway and other oxidative stress-related genes points to a potential oxidative imbalance, which may further influence starch metabolism through redox regulation. These findings provide insights into the metabolic interconnections between starch accumulation and cytosolic acetyl-CoA metabolism, highlighting the complex regulatory networks that coordinate plant growth and development. Future studies should explore these interactions in greater detail to elucidate the mechanisms driving these metabolic changes.

## Figures and Tables

**Table 1:** Pathways with up- or down-regulated genes in 42-DAI seedlings of antisense-*ACLA* mutant grown in an SD diurnal cycle. Parentheses indicate the number of genes with differentially accumulated mRNA in a pathway compared to the total number of genes in that pathway. Pathways are from MetNetDB [55].

Pathways with Up-Regulated Genes (41/173)	Pathways with Down-Regulated Genes (71/227)
***Cell wall metabolism (7/24)***	***Cell wall metabolism (17/42)***
Xylose degradation (1/3)	Mannose degradation (1/2)
Xylulose-monophosphate cycle (1/4)	GDP-D-rhamnose biosynthesis (5/16)
D-arabinose catabolism (1/4)	(Deoxy)ribose phosphate metabolism (4/10)
Dolichyl-diphosphooligosaccharide biosynthesis (2/10)	Glucosinolate biosynthesis from homomethionine (2/5)
Sinapate ester biosynthesis (2/3)	Monoterpene biosynthesis (2/3)
***Fatty acid elongation (12/32)***	Phenylpropanoid biosynthesis (3/6)
Fatty acid elongation (3/5)	***Fatty acid oxidation (2/17)***
Fatty acid omega-oxidation (1/2)	Fatty acid beta-oxidation (2/17)
Cutin biosynthesis (1/1)	***Sulfur containing amino acid biosynthesis (17/63)***
Mannosyl-chito-dolichol biosynthesis (2/3)	Sulfur assimilation (6/21)
Mevalonate pathway (5/21)	Homocysteine and cysteine interconversion (3/8)
***Amino acid metabolism (8/33)***	Cysteine biosynthesis (2/13)
Phenylalanine biosynthesis I (2/3)	Methionine biosynthesis (3/12)
Tyrosine biosynthesis II (2/4)	Threonine biosynthesis from homoserine (3/9)
Lysine biosynthesis (2/19)	***Hormone metabolism and signal transduction (27/74)***
Serine biosynthesis (2/7)	Jasmonic acid biosynthesis (5/18)
***Hormone metabolism (2/8)***	Abscisic acid biosynthesis (4/6)
Brassinosteroid biosynthesis (2/8)	Cytokinins degradation (3/6)
***Vitamins (3/11)***	Brassinosteroids signaling (4/12)
Biotin biosynthesis (3/11)	APX1 signal transduction pathway (5/13)
***Other (9/65)***	Lipoxygenase pathway (4/11)
Chlorophyll Biosynthesis (3/17)	Autophagy (2/8)
Glyoxylate cycle (2/17)	***Other (8/31)***
Glucose 1-phosphate metabolism (1/4)	Glutamate degradation III (1/3)
De novo biosynthesis of pyrimidine ribonucleotides (3/27)	Tryptophan biosynthesis (7/28)

**Table 2:** Differentially expressed carbohydrate- and cell wall-metabolism genes in the antisense-*ACLA* mutant. Fold-change values (shown in bold) were calculated by comparing expression of each gene in the WT plants at three time points: 1 h in the light (L1); and 0.5 h (D0.5) and 4 h (D4) in the dark. Only genes with greater than 2-fold changes in values, and with error bars that do not overlap between genotypes, are listed. Expression in antisense-*ACLA* mutant is up-regulated (red fold-change) or down-regulated (blue fold-change).

Genes Up-Regulated	Genes Down-Regulated
***Starch synthesis***	***Starch degradation***
ADPGlu-PP (*APL4*, At4g39210) **2** (L1)	*AAM2* (At4g25000)**-2** (L1)
ADPGlu-PP (*APL3*, At2g21590) **3.4 ** (D0.5)	*BAM1* (At4g15210)**-3** (D0.5)
***Trehalose***	***Trehalose***
Trehalose-6-P phosphatase (At5g65140) **3** (L1)	Trehalose-6-P phosphatase (At1g22210)**-3** (L1, D4)
Trehalose-6-P phosphatase (At4g12430) **2.1** (D0.5)	
***Cell wall synthesis***	***Cell wall synthesis***
UDP-galactose 4-epimerase (At2g34850) **6.2** (D0.5)	UDP-glucose/galactose 4-epimerase-like protein (At4g20460)**-2** (D0.5)
Cellulose synthase like gene (*AtCslA10*, At1g24070) **3.2** (D0.5)	Mannose-6-phosphate isomerase 1 (At3g02570)**-2** (D0.5)
Cellulose synthase like gene (*AtCslA03*, At1g23480) **2** (D4)	Rhamnosyltransferase, hemicellulose (At1g64910)**-2** (D0.5)
***Cell wall modification***	***Cell wall modification***
Beta-expansin (*EXPB1*, At2g20750) **2** (L1)	Xyloglucan endotransglycosylase (At5g65730)**-2** (L1)
Beta-expansin (*EXPB3*, At4g28250) **3** (L1) **2.4** (D0.5)	Pectinesterase (At2g26440)**-3** (D0.5)
Invertase/pectin methylesterase inhibitor (At2g47670) **3.7** (D0.5)	Pectinesterase (At4g33220)**-2** (D0.5)
Expansin, putative (*EXP9*, At5g02260) **3** (D4)	Pectinesterase (At2g47550)**-2** (D4)
Xyloglucan endotransglycosylase 1 (At3g23730) **5** (D4)	Pectinacetylesterase (At3g09410)**-2** (D4)
Xyloglucan endotransglycosylase (At4g03210) **5** (D4)	Invertase/pectin methylesterase inhibitor (At1g10770)**-3** (D4)
Xyloglucan endotransglycosylase (At1g10550) **4** (D4)	
Endo-xyloglucan transferase (*XTR7*) (At4g14130) **3** (D4)	
Pectin methylesterase (At5g47500) **2** (D4)	
Invertase/pectin methylesterase inhibitor (At3g47380) **2** (D4)	
***Cell wall degradation***	***Cell wall degradation***
(1-4)-beta-mannan endohydrolase (At5g66460) **3** (L1)	Xyloglucan endo-1,4-beta-D-glucanase (*SEN4*, At4g30270)**-4** (L1)
Polygalacturonase (At1g10640) **3** (L1)	Xyloglucan endo-1,4-beta-D glucanase (*XTR-3*, At4g30280)**-3** (L1)
Pectate lyase (At1g67750) **2** (L1) **2.1 ** (D0.5)	Pectate lyase (At4g13210)**-2** (L1)
Beta-xylosidase (At1g02640) **2** (D4)	Polygalacturonase inhibiting protein 2 (*PGIP2*, At5g06870)**-2** (D0.5)
	Xyloglucan endo-1,4-beta-D-glucanase (*XTR-6*, At4g25810)**-2** (D0.5)
	Xyloglucan endo-1,4-beta-D-glucanase (At5g48070)**-2** (D4)

**Table 3:** Genes that are highly correlated with *ISA1* expression at the level of RNA accumulation, as analyzed by MOG [58].

Gene	Correlation Coefficient	Locus ID
*ISA1*	1.00	At2g39930
*ISA2*	0.74	At1g03310
*Pti1*	0.73	At1g26150
*DPE1*	0.72	At5g64860
Expressed protein	0.72	At4g10470
*RCP1*/*MEX1*	0.68	At5g17520
Desulfhydrase	0.67	At1g48420
Expressed protein	0.67	At3g60810
*PHS1*	0.66	At3g29320

## Data Availability

All relevant data are within the manuscript and its Supporting Information files. Accession numbers: Sequence data from this article can be found under the following accession numbers in The Arabidopsis Genome Information Resource: *ACLA1* (At1g10670) (access date: 2 October 2024). GeneChip data presented in this publication were deposited in NCBI’s Gene Expression Omnibus and are accessible through GEO Series accession number GSE254150.

## Supplementary Materials

The following supporting information can be downloaded at: https://www.mdpi.com/article/10.3390/ijms251910850/s1, Refs. [12,13,15,20,39,41,52,97,98,99,100,101,102,103,104,105,106,107,108,109,110,111,112,113,114,115,116,117,118] are included in Supplementary Materials.
