# Supplementary material for: Modulation of the Arabidopsis Starch Metabolic Network by the Cytosolic Acetyl-CoA Pathway in the Context of the Diurnal Illumination Cycle
Source: Int J Mol Sci. 2024 Oct 9;25(19):10850. doi: 10.3390/ijms251910850 (PMC11477042; doi:10.3390/ijms251910850)
Supplement: Supplementary file 1 [file ijms-25-10850-s001.zip › ijms-3235139-supplementary.pdf]

Supplementary materials for

Modulation of the Arabidopsis starch metabolic network by the cytosolic acetyl-CoA pathway in the context of the diurnal illumination cycle

Lei Wang [1,2,†], Carol M. Foster [3,†], Wieslawa I. Mentzen [3], Rezwan Tanvir [2], Yan Meng [4], Basil J. Nikolau [5,6], Dan Nettleton [7], Eve Syrkin Wurtele [3,6,*], and Ling Li [2,*].

[1] College of Life Sciences, Shihezi University, Shihezi, 832003, China; wangleibailu@163.com (L.W.)

[2] Department of Biological Sciences, Mississippi State University, Mississippi State, MS, 39762, USA; rt916@msstate.edu (R.T.)

[3] Department of Genetics, Development and Cell Biology, Iowa State University, Ames, IA, 50011, USA; cmfoster@cox.net (C.M.F.); wimentzen@gmail.com (W.I.M.)

[4] Department of Agriculture, Alcorn State University, Lorman, MS, 39096, USA; ymeng@alcorn.edu (Y.M.)

[5] Roy J. Carver Department of Biochemistry, Biophysics, and Molecular Biology, Iowa State University, Ames, IA, 50011, USA; dimmas@iastate.edu (B.J.N.)

[6] Center for Metabolic Biology, Iowa State University, Ames, IA, 50011, USA

[7] Department of Statistics, Iowa State University, Ames, IA, 50011, USA; dnett@iastate.edu (D.N.)

[*] Correspondence: liling@biology.msstate.edu (L.L.); evewurtele@gmail.com (E.S.W.)

[†] These authors have contributed equally to this work

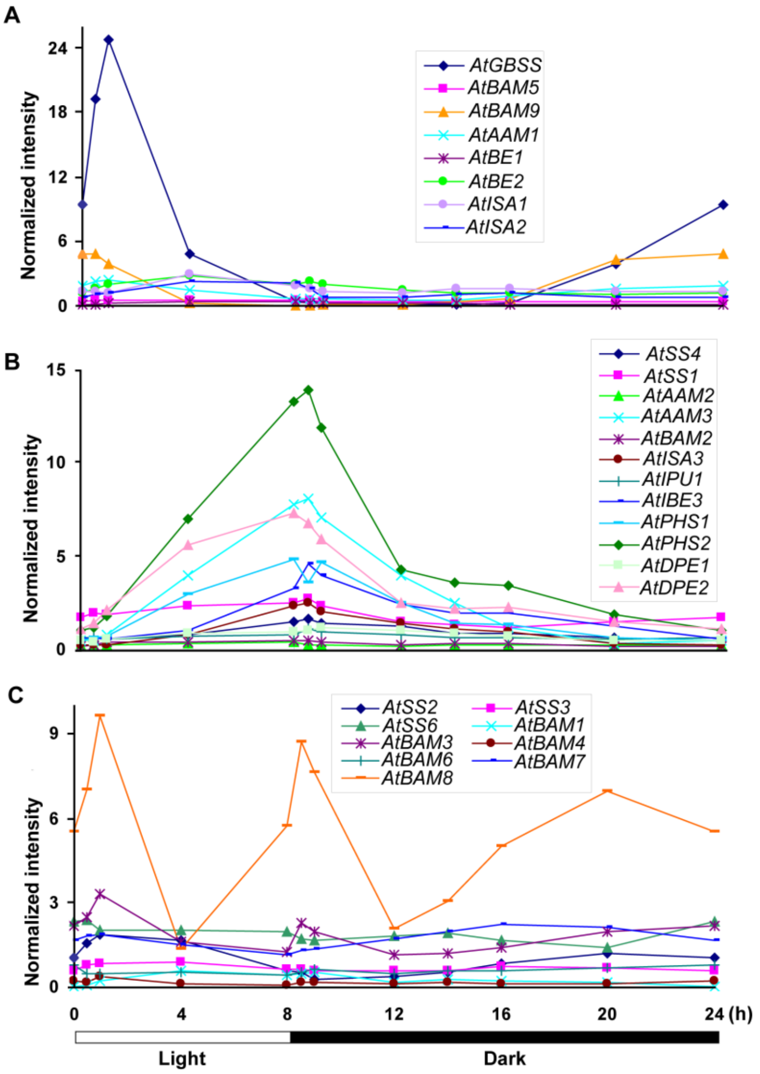

Supplementary Figure S1. mRNAs of genes encoding enzymes involved in starch metabolism accumulate in three overall patterns: (**A**) peak in the light, (**B**) peak in the dark, (**C**) peak in both the light and the dark. Overall expression profiles of starch metabolic genes in WT over the SD diurnal cycle. The average normalized intensity for each microarray chip is 1.

Supplementary Table S1. Genes with the most significantly changes in transcript levels over the SD diurnal cycle in WT plants (FDR controlled at 0.00031). Supplement to Figure 3. Carbohydrate metabolic genes, genes involved in transcription, genes responded to cold.

Cluster 1

Locus ID	p-value	q-value	Annotation	MapMan Category
AT4G22570	3.30E-06	0.0002876	adenine phosphoribosyltransferase	nucleotide metabolism.salvage
AT1G08460	5.46E-08	3.36E-05	histone deacetylase family protein	RNA.regulation of transcription.HDA
AT1G05890	7.59E-07	0.0001203	zinc finger protein	RNA.regulation of transcription.unclassified
AT1G49200	9.76E-07	0.0001345	zinc finger (C3HC4-type RING finger) family protein	RNA.regulation of transcription.unclassified
AT3G17590	3.54E-06	0.000305	transcription regulatory protein SNF5	RNA.regulation of transcription.Chromatin Remodeling Factors
AT5G35330	3.23E-06	0.000287	methyl-CpG-binding domain-containing protein	RNA.regulation of transcription.Methyl binding domain proteins
AT5G22000	5.80E-07	0.0001053	zinc finger (C3HC4-type RING finger)	RNA.regulation of transcription.unclassified
AT1G47270	3.12E-07	8.06E-05	F-box family protein / tubby family protein	protein.degradation.ubiquitin.E3.SCF.FBOX; cell.organisation
AT4G27130	7.06E-07	0.0001164	Translation initiation factor SUI1	protein.synthesis.initiation
AT1G64040	9.69E-07	0.0001345	phosphoprotein Ser/Thr phosphatase	protein.postranslational modification
AT2G25070	2.12E-06	0.0002243	protein phosphatase 2C	protein.postranslational modification
AT3G02740	8.29E-07	0.0001225	aspartyl protease	protein.degradation.aspartate protease
AT5G17290	1.01E-07	4.17E-05	Autophagy protein ATG5	protein.degradation.autophagy
AT1G69295	5.51E-07	0.0001027	beta-1,3-glucanase-related	misc.beta 1,3 glucan hydrolases
AT1G47960	2.24E-06	0.0002326	invertase/pectin methylesterase inhibitor	cell wall.pectin*esterases.PME; misc.invertase/pectin methylesterase inhibitor family protein
AT4G14900	4.66E-07	9.72E-05	hydroxyproline-rich glycoprotein	cell wall.cell wall proteins.HRGP
AT4G15780	1.10E-08	1.39E-05	synaptobrevin-related	cell. vesicle transport
AT4G32150	1.35E-06	0.0001678	synaptobrevin family protein	cell. vesicle transport
AT2G30540	3.06E-06	0.0002776	GLUTAREDOXIN	redox.glutaredoxins
AT3G01520	1.30E-06	0.0001661	universal stress protein	stress.abiotic.unspecified
AT1G75750	3.29E-06	0.0002876	gibberellin-regulated protein 1 (GASA1)	hormone metabolism.gibberelin.induced-regulated-responsive-activated
AT1G05810	1.20E-06	0.000156	Ras-related protein (ARA-1)	signalling.G-proteins
AT1G16920	2.21E-06	0.0002316	Ras-related GTP-binding protein	signalling.G-proteins
AT1G74880	6.42E-07	0.000109	Ndh complex	not assigned.unknown
AT2G39000	6.21E-07	0.000109	N-acetyltransferase	not assigned.no ontology
AT3G54120	9.74E-07	0.0001345	reticulon family protein	not assigned.no ontology
AT5G56750	3.42E-07	8.22E-05	Ndr family protein	not assigned.no ontology
AT1G30880	1.62E-06	0.0001919	expressed protein	not assigned.unknown
AT1G68440	8.11E-07	0.0001225	expressed protein	not assigned.unknown
AT3G05760	1.79E-06	0.0002046	expressed protein	not assigned.unknown
AT4G18740	1.17E-06	0.0001529	expressed protein	not assigned.unknown
AT3G55420	2.12E-06	0.0002243	expressed protein	not assigned.unknown
AT5G11950	2.60E-06	0.000256	expressed protein	not assigned.unknown
AT5G42680	9.09E-10	6.26E-06	expressed protein	not assigned.unknown
AT1G58032	3.08E-06	0.0002776	unknown	

Cluster 2

Locus ID	p-value	q-value	Annotation	MapMan Category
AT1G70000	4.93E-07	9.78E-05	DNA-binding family protein	RNA.regulation of transcription.MYB-related transcription factor family
AT2G20570	7.67E-07	0.0001203	golden2-like transcription factor	RNA.regulation of transcription.G2-like transcription factor family, GARP
AT3G09600	2.12E-09	6.89E-06	myb family transcription factor	RNA.regulation of transcription.MYB-related transcription factor family

AT3G02830	6.79E-08	3.59E-05	zinc finger (CCCH-type) family protein	RNA.regulation of transcription.C2H2 zinc finger family
AT4G00050	4.84E-08	3.18E-05	basic helix-loop-helix (bHLH) family protein	RNA.regulation of transcription.bHLH,Basic Helix-Loop-Helix family
AT5G02840	3.36E-09	7.64E-06	myb family transcription factor	RNA.regulation of transcription.MYB-related transcription factor family
AT5G44190	9.94E-07	0.0001362	myb family transcription factor (GLK2)	RNA.regulation of transcription.G2-like transcription factor family, GARP
AT5G15830	1.05E-06	0.000143	bZIP transcription factor family protein	RNA.regulation of transcription.bZIP transcription factor family
AT5G15850	2.99E-06	0.0002752	zinc finger protein CONSTANS-LIKE 1	RNA.regulation of transcription.C2C2(Zn) CO-like, Constans-like zinc finger family; development.unspecified
AT2G15580	1.31E-06	0.0001665	zinc finger (C3HC4-type RING finger) family protein	RNA.regulation of transcription.C3H zinc finger family
AT1G60000	1.25E-06	0.0001615	ribonucleoprotein	RNA.regulation of transcription.unclassified; RNA.RNA binding
AT4G27000	7.66E-07	0.0001203	RNA-binding protein 45	RNA.regulation of transcription.unclassified
AT5G28020	4.11E-08	2.83E-05	cysteine synthase	amino acid metabolism.synthesis.serine-glycine-cysteine group.cysteine.OASTL
AT2G16500	6.22E-07	0.000109	arginine decarboxylase	amino acid metabolism.degradation.glutamate family.arginine; polyamine metabolism
AT5G27360	9.55E-07	0.0001345	sugar-porter family protein 2	transporter.sugars
AT2G47490	1.65E-09	6.26E-06	mitochondrial substrate carrier family protein	transport.metabolite transporters at the mitochondrial membrane
AT3G21390	4.23E-07	9.16E-05	mitochondrial substrate carrier family protein	transport.metabolite transporters at the mitochondrial membrane
AT5G47560	3.54E-07	8.22E-05	malate/fumarate transporter.	transport.unspecified cations
AT2G26690	9.20E-10	6.26E-06	nitrate transporter	transport.nitrate
AT1G33110	5.40E-07	0.0001024	MATE efflux family protein	transport misc
AT5G49730	1.51E-07	4.98E-05	ferric chelate reductase	metal handling.acquisition
AT4G25700	1.07E-06	0.000144	beta-carotene hydroxylase	secondary metabolism.isoprenoids.carotenoids
AT1G64500	7.63E-08	3.84E-05	glutaredoxin family protein	redox.glutaredoxins
AT1G52540	2.21E-07	6.36E-05	protein kinase	signalling.receptor kinases; development.unspecified
AT1G62960	1.65E-07	5.29E-05	1-aminocyclopropane-1-carboxylate synthase	hormone metabolism.ethylene.synthesis-degradation
AT3G14770	3.60E-06	0.0003055	nodulin MtN3 family protein	development.unspecified
AT1G07010	2.13E-06	0.0002243	calcineurin-like phosphoesterase family protein	misc.calcineurin-like phosphoesterase family protein
AT1G13080	3.56E-08	2.61E-05	cytochrome P450 family protein	misc.cytochrome P450
AT4G37550	1.35E-06	0.0001678	formamidase	misc.misc2
AT3G25585	1.05E-07	4.17E-05	aminoalcoholphosphotransferase	misc.misc2
AT2G41250	1.18E-07	4.19E-05	Haloacid dehalogenase-like hydrolase	not assigned.no ontology
AT2G19650	5.17E-07	9.97E-05	DC1 domain-containing protein	not assigned.no ontology
AT4G25830	1.09E-07	4.17E-05	integral membrane family protein	not assigned.no ontology
AT5G62130	9.50E-07	0.0001345	Per1-like protein-related	not assigned.no ontology
AT4G26850	1.42E-08	1.70E-05	ascorbate biosynthesis	not assigned.unknown
AT5G19290	3.59E-06	0.0003055	esterase/lipase/thioesterase family protein	not assigned.no ontology
AT4G26860	3.93E-09	8.13E-06	alanine racemase family protein	not assigned.no ontology
AT3G11620	1.34E-06	0.0001678	expressed protein	not assigned.unknown
AT3G60910	3.51E-07	8.22E-05	expressed protein	not assigned.no ontology
AT1G62250	2.88E-06	0.0002718	expressed protein	not assigned.unknown
AT2G04039	2.53E-06	0.0002523	expressed protein	not assigned.unknown
AT1G29700	2.54E-06	0.0002523	expressed protein	not assigned.unknown
AT3G15310	5.03E-09	8.80E-06	expressed protein	not assigned.unknown
AT1G75180	1.38E-07	4.62E-05	expressed protein	not assigned.unknown
AT2G33250	2.51E-06	0.0002515	expressed protein	not assigned.unknown
AT4G24700	1.76E-07	5.48E-05	expressed protein	not assigned.unknown
AT5G18040	1.28E-06	0.0001645	expressed protein	not assigned.unknown

Cluster 3

Locus ID	p-value	q-value	Annotation	MapMan Category
AT2G21320	3.23E-07	8.16E-05	zinc finger (B-box type) family protein	RNA.regulation of transcription.C2C2(Zn) CO-like, Constans-like zinc finger family
AT2G31380	2.40E-07	6.71E-05	B-box zinc finger protein	RNA.regulation of transcription.C2C2(Zn) CO-like, Constans-like zinc finger family
AT1G01060	4.45E-07	9.48E-05	myb family transcription factor	RNA.regulation of transcription.MYB-related transcription factor family
AT3G02380	7.55E-07	0.0001203	zinc-finger protein	RNA.regulation of transcription.C2C2(Zn) CO-like, Constans-like zinc finger family; development.unspecified
AT3G21890	1.51E-06	0.0001837	zinc finger (B-box type) family protein	RNA.regulation of transcription.C2C2(Zn) CO-like, Constans-like zinc finger family
AT4G38960	2.22E-06	0.0002316	zinc finger (B-box type) family protein	RNA.regulation of transcription.C2C2(Zn) CO-like, Constans-like zinc finger family
AT5G59780	8.14E-07	0.0001225	myb family transcription factor (MYB59)	RNA.regulation of transcription.MYB domain transcription factor family
AT2G26800	6.27E-07	0.000109	hydroxymethylglutaryl-CoA lyase	amino acid metabolism.degradation. branched-chain group.leucine; secondary metabolism.isoprenoids.mevalonate pathway
AT1G55920	3.41E-06	0.0002949	Encodes a chloroplast/cytosol localized serine O-acetyltransferase	amino acid metabolism.synthesis.serine-glycine-cysteine group.cysteine.SAT; metal handling.binding, chelation and storage
AT3G08730	1.46E-06	0.0001795	protein-serine kinase	protein.synthesis.misc ribososomal protein
AT5G15950	3.26E-08	2.61E-05	adenosylmethionine decarboxylase	polyamine metabolism
AT5G44530	8.33E-07	0.0001225	subtilase family protein	protein.degradation.subtilases
AT2G22990	4.86E-07	9.78E-05	sinapoylglucose:malate sinapoyltransferase	protein.degradation.serine protease
AT2G27420	4.79E-07	9.78E-05	cysteine proteinase	protein.degradation.cysteine protease
AT2G24540	2.64E-06	0.0002566	kelch repeat-containing F-box family protein	protein.degradation.ubiquitin.E3.SCF.FBOX
AT5G47970	2.54E-07	6.96E-05	nitrogen regulation family protein	N-metabolism.misc
AT1G07180	3.84E-08	2.73E-05	NAD(P)H dehydrogenase	mitochondrial electron transport / ATP synthesis.NADH-DH.type II.internal matrix
AT1G80760	2.13E-07	6.21E-05	major intrinsic family protein	transport.Major Intrinsic Proteins.NIP
AT3G01550	3.26E-09	7.64E-06	AtPPT2	transport.metabolite transporters at the envelope membrane
AT1G19450	2.52E-08	2.39E-05	integral membrane protein	transporter.sugars
AT3G11900	1.95E-06	0.0002134	amino acid transporter family protein	transport.amino acids
AT5G64840	1.75E-06	0.0002021	ABC transporter family protein	transport.ABC transporters and multidrug resistance systems
AT2G46800	1.06E-06	0.0001435	member of Zinc transporter (ZAT) family	transport.metal
AT1G58290	7.23E-08	3.74E-05	glutamyl-tRNA reductase	tetrapyrrole synthesis
AT1G44446	2.88E-07	7.53E-05	chlorophyll a oxygenase (CAO) / chlorophyll b synthase	tetrapyrrole synthesis
AT5G64930	7.59E-07	0.0001203	CPR5 protein	stress.biotic
AT1G65870	4.00E-07	8.92E-05	disease resistance-responsive family protein	stress.biotic
AT5G67030	2.72E-07	7.28E-05	zeaxanthin epoxidase	hormone metabolism.abscisic acid.synthesis-degradation
AT5G54130	1.08E-07	4.17E-05	calcium-binding EF hand family protein	signalling.calcium
AT1G32900	3.36E-09	7.64E-06	GBSS	major CHO metabolism.synthesis. starch.starch synthase
AT2G22240	6.48E-08	3.59E-05	inositol-3-phosphate synthase isozyme 2	minor CHO metabolism.myo-inositol.InsP Synthases
AT1G78510	1.98E-06	0.0002134	solanesyl diphosphate synthase (SPS)	secondary metabolism. isoprenoids. terpenoids
AT1G07450	9.60E-07	0.0001345	tropinone reductase	secondary metabolism.N misc.alkaloid-like; misc.nitrilases, *nitrile lyases, berberine bridge enzymes, reticuline oxidases, troponine reductases
AT2G34460	2.37E-08	2.34E-05	flavin reductase-related	not assigned.no ontology

AT4G39510	1.37E-06	0.0001694	cytochrome P450 family protein	misc.cytochrome P450
AT3G17040	3.00E-08	2.61E-05	RNA tetratricopeptide repeat-containing protein	not assigned.no ontology.pentatricopeptide (PPR) repeat-containing protein
AT2G41010	1.81E-06	0.0002048	VQ motif-containing protein	not assigned.no ontology
AT1G17870	6.38E-07	0.000109	S2P-like putative metalloprotease	not assigned.unknown
AT4G15550	2.11E-07	6.21E-05	UDP-glucose:indole-3-acetate beta-D-glucosyltransferase	not assigned.no ontology
AT3G61080	5.89E-08	3.37E-05	fructosamine kinase family protein	not assigned.no ontology
AT5G43850	8.35E-07	0.0001225	acireductone dioxygenase	not assigned.no ontology
AT5G15910	2.25E-06	0.0002326	dehydrogenase-related	not assigned.no ontology
AT5G20380	2.01E-06	0.0002157	transporter-related	not assigned.no ontology
AT2G41120	1.52E-08	1.73E-05	expressed protein	not assigned.unknown
AT2G36630	2.94E-06	0.0002741	expressed protein	not assigned.unknown
AT1G22850	2.69E-06	0.0002593	expressed protein	not assigned.unknown
AT1G55960	2.38E-06	0.0002417	expressed protein	not assigned.no ontology
AT1G69160	8.22E-07	0.0001225	expressed protein	not assigned.unknown
AT3G12320	3.00E-06	0.0002752	expressed protein	not assigned.unknown
AT1G16730	4.45E-09	8.43E-06	expressed protein	not assigned.unknown
AT4G17840	1.02E-07	4.17E-05	expressed protein	not assigned.unknown
AT4G27030	1.08E-06	0.0001445	expressed protein	not assigned.unknown
AT3G54500	1.90E-06	0.0002108	expressed protein	not assigned.unknown
AT3G56290	1.68E-06	0.000196	expressed protein	not assigned.unknown
AT5G43180	1.53E-06	0.0001841	expressed protein	not assigned.unknown
AT5G54120	7.76E-08	3.84E-05	expressed protein	not assigned.unknown
AT5G59400	6.97E-07	0.0001157	expressed protein	not assigned.unknown
AT5G61670	8.10E-07	0.0001225	expressed protein	not assigned.unknown
AT5G12470	3.33E-07	8.22E-05	expressed protein	not assigned.unknown

Cluster 4

Locus ID	p-value	q-value	Annotation	MapMan Category
AT5G35970	1.89E-07	5.66E-05	DNA-binding protein	DNA.unspecified
AT1G06040	1.12E-06	0.000149	B-box zinc finger	RNA.regulation of transcription.C2C2(Zn) CO-like, Constans-like zinc finger family
AT4G18390	2.94E-06	0.0002741	TCP family transcription factor	RNA.regulation of transcription.TCP transcription factor family
AT4G30960	3.07E-06	0.0002776	CBL-interacting protein kinase 6	protein.postranslational modification
AT5G45800	1.97E-06	0.0002134	leucine-rich repeat transmembrane protein kinase	protein.postranslational modification
AT5G02180	8.62E-08	4.00E-05	amino acid transporter family protein	transport.amino acids
AT2G29650	6.72E-08	3.59E-05	inorganic phosphate transporte	transport.phosphate
AT4G13800	8.07E-07	0.0001225	permease-related	transport misc
AT2G05070	1.16E-07	4.19E-05	chlorophyll A-B binding protein	PS.lightreaction.photosystem II.LHC-II
AT2G36870	3.09E-06	0.0002776	xyloglucan:xyloglucosyl transferase	cell wall.modification
AT1G15950	9.27E-08	4.17E-05	cinnamoyl CoA reductase	secondary metabolism.phenylpropanoids.lignin biosynthesis.CCR1
AT2G39980	1.84E-06	0.0002072	transferase	secondary metabolism.flavonoids.anthocyanins
AT4G32770	2.62E-06	0.0002566	Tocopherol cyclase	secondary metabolism. isoprenoids. tocopherol biosynthesis
AT3G57020	4.84E-07	9.78E-05	strictosidine synthase	secondary metabolism.N misc.alkaloid-like
AT4G13010	8.06E-08	3.90E-05	oxidoreductase	misc.oxidases - copper, flavone etc.
AT1G10370	1.75E-06	0.0002021	glutathione S-transferase	misc.glutathione S transferases
AT3G48460	7.73E-07	0.0001204	GDSL-motif lipase/hydrolase family protein	misc.GDSL-motif lipase
AT5G42250	9.12E-07	0.0001305	alcohol dehydrogenase	misc.alcohol dehydrogenases
AT1G18360	2.28E-06	0.0002336	hydrolase	not assigned.no ontology
AT1G73480	3.06E-08	2.61E-05	hydrolase, alpha/beta fold family protein	not assigned.no ontology
AT2G40400	2.85E-07	7.53E-05	expressed protein	not assigned.unknown

Cluster 5

Locus ID	p-value	q-value	Annotation	MapMan Category
AT3G57150	2.67E-06	0.0002584	dyskerin	DNA.synthesis/chromatin structure
AT4G04840	1.51E-06	0.0001837	methionine sulfoxide reductase domain-containing protein / SeIR domain-containing protein	RNA.regulation of transcription.putative DNA-binding protein
AT3G56330	1.16E-06	0.0001529	N2,N2-dimethylguanosine tRNA methyltransferase	RNA.regulation of transcription.unclassified
AT4G39260	7.31E-07	0.0001191	glycine-rich RNA binding protein CCR1	RNA.regulation of transcription.GRP
AT1G11790	3.00E-06	0.0002752	prephenate dehydratase family protein	amino acid metabolism.synthesis.aromatic aa.phenylalanine
AT2G31610	3.06E-06	0.0002776	40S ribosomal protein S3 (RPS3A)	protein.synthesis.misc ribososomal protein
AT4G02930	3.71E-07	8.52E-05	elongation factor Tu	protein.synthesis.elongation
AT4G33490	3.97E-07	8.92E-05	nucellin protein	protein.degradation
AT1G69830	9.04E-07	0.0001301	AAM3	major CHO metabolism.degradation.starch.starch cleavage
AT1G22650	3.21E-06	0.0002863	beta-fructofuranosidase	major CHO metabolism.degradation.sucrose.misc
AT5G26340	3.23E-07	8.16E-05	hexose transporter	transporter.sugars
AT1G22570	5.31E-08	3.36E-05	proton-dependent oligopeptide transport	transport.peptides and oligopeptides
AT5G62360	1.57E-06	0.000188	invertase/pectin methylesterase inhibitor family protein, INV	misc.invertase/pectin methylesterase inhibitor family protein
AT5G03350	3.32E-06	0.0002882	legume lectin family protein	misc.myrosinases-lectin-jacalin
AT2G42530	9.63E-09	1.29E-05	cold-responsive protein / cold-regulated protein (cor15b)	stress.abiotic.cold
AT1G20450	2.97E-06	0.0002752	dehydrin (ERD10)	stress.abiotic.unspecified
AT1G56300	1.09E-07	4.17E-05	DNAJ heat shock N-terminal domain-containing protein	stress.abiotic.heat
AT5G47220	2.83E-06	0.0002693	ERF (ethylene response factor) subfamily B-3 of ERF/AP2 transcription factor family	hormone metabolism.ethylene.signal transduction
AT3G07390	1.88E-06	0.0002096	auxin-responsive protein	hormone metabolism.auxin.induced-regulated-responsive-activated
AT3G16050	3.28E-06	0.0002876	stress-responsive protein	hormone metabolism.ethylene.induced-regulated-responsive-activated
AT3G44860	2.63E-06	0.0002566	S-adenosyl-L-methionine:carboxyl methyltransferase	hormone metabolism.salicylic acid.synthesis-degradation
AT5G39790	1.81E-06	0.0002048	5'-AMP-activated protein kinase beta-1 subunit-related	signalling.in sugar and nutrient physiology
AT5G45750	3.12E-08	2.61E-05	Ras-related GTP-binding protein	signalling.G-proteins
AT2G45560	3.56E-08	2.61E-05	cytochrome P450 family protein	misc.cytochrome P450
AT2G34260	5.69E-08	3.37E-05	transducin family protein	not assigned.no ontology
AT1G76590	3.27E-06	0.0002876	zinc-binding family protein	not assigned.no ontology
AT3G17170	9.62E-09	1.29E-05	ribosomal protein S6 family protein	not assigned.no ontology
AT3G18760	7.51E-09	1.21E-05	ribosomal protein S6 family protein	not assigned.no ontology
AT3G27280	6.28E-07	0.000109	prohibitin	not assigned.no ontology
AT4G34120	5.77E-07	0.0001053	CBS domain-containing protein	not assigned.no ontology
AT5G38890	2.74E-06	0.000263	exoribonuclease-related	not assigned.no ontology
AT5G47240	4.96E-07	9.78E-05	MutT/nudix family protein	not assigned.no ontology
AT5G19750	1.03E-07	4.17E-05	peroxisomal membrane 22 kDa family protein	not assigned.no ontology
AT1G75860	1.66E-06	0.0001946	expressed protein	not assigned.unknown
AT2G22870	2.62E-07	7.09E-05	expressed protein	not assigned.unknown
AT2G38790	1.81E-07	5.49E-05	expressed protein	not assigned.unknown
AT1G10522	4.46E-07	9.48E-05	expressed protein	not assigned.unknown
AT1G28395	4.59E-07	9.67E-05	expressed protein	not assigned.unknown
AT1G48460	4.10E-07	9.05E-05	expressed protein	not assigned.unknown
AT1G20220	2.50E-06	0.0002515	expressed protein	RNA.regulation of transcription.putative DNA-binding protein
AT1G27930	5.48E-07	0.0001027	expressed protein	not assigned.unknown
AT3G15000	3.54E-07	8.22E-05	expressed protein	not assigned.no ontology
AT4G24175	7.33E-07	0.0001191	expressed protein	not assigned.unknown

AT5G47455	2.30E-06	0.0002346	expressed protein	not assigned.unknown

Cluster 6

Locus ID	p-value	q-value	Annotation	MapMan Category
AT1G34380	7.95E-09	1.21E-05	5'-3' exonuclease family protein	DNA.synthesis/chromatin structure
AT3G27360	3.27E-06	0.0002876	histone H3	DNA.synthesis/chromatin structure.histone
AT5G10390	2.44E-06	0.0002467	histone H3	DNA.synthesis/chromatin structure.histone
AT1G05805	1.23E-07	4.30E-05	basic helix-loop-helix (bHLH) family protein	RNA.regulation of transcription.bHLH,Basic Helix-Loop-Helix family
AT1G49230	1.10E-07	4.17E-05	zinc finger (C3HC4-type RING finger) family protein	RNA.regulation of transcription.unclassified
AT1G19310	3.38E-07	8.22E-05	zinc finger (C3HC4-type RING finger) family protein	RNA.regulation of transcription.C3H zinc finger family
AT5G50450	2.09E-06	0.0002232	zinc finger (MYND type) family protein	RNA.regulation of transcription.unclassified
AT3G53460	8.90E-07	0.0001289	29 kDa ribonucleoprotein	RNA.regulation of transcription.unclassified; RNA.RNA binding
AT2G21660	4.71E-11	1.07E-06	glycine-rich RNA-binding protein (GRP7)	RNA.RNA binding
AT3G22200	1.80E-07	5.49E-05	4-aminobutyrate aminotransferase	amino acid metabolism.synthesis.central amino acid metabolism.GABA
AT2G28900	2.28E-06	0.0002336	mitochondrial import inner membrane translocase subunit	transport.protein transport mitochondrial membrane (TIM/TOM)
AT4G13510	1.08E-07	4.17E-05	ammonium transporter	transport.ammonium
AT4G26670	1.13E-07	4.19E-05	mitochondrial import inner membrane translocase subunit	transport.protein transport mitochondrial membrane (TIM/TOM)
AT5G11150	1.66E-06	0.0001946	synaptobrevin / vesicle-associated membrane protein	cell. vesicle transport
AT1G76790	8.60E-07	0.0001254	O-methyltransferase	secondary metabolism.simple phenols
AT4G38580	1.52E-06	0.0001839	heavy-metal-associated domain-containing protein	metal handling.binding, chelation and storage
AT5G06690	6.36E-07	0.000109	thioredoxin family protein	redox.thioredoxin
AT3G26740	1.17E-06	0.0001529	transcripts are differentially regulated at the level of mRNA stability at different times of day controlled by the circadian clock	signalling.light
AT2G02100	1.51E-09	6.26E-06	plant defensin-fusion protein	stress.biotic
AT1G53350	2.59E-06	0.000256	disease resistance protein	stress.biotic
AT2G43530	3.60E-06	0.0003055	defensin-like (DEFL) family protein	stress.biotic
AT4G30650	1.18E-07	4.19E-05	low temperature and salt responsive protein	stress.abiotic.cold; stress.abiotic.drought/salt
AT4G30660	4.84E-07	9.78E-05	low temperature and salt responsive protein	stress.abiotic.cold; stress.abiotic.drought/salt
AT4G13850	1.97E-06	0.0002134	glycine-rich RNA-binding protein (GRP2)	stress.abiotic.cold; RNA.regulation of transcription.unclassified; RNA.RNA binding
AT2G33830	1.27E-09	6.26E-06	dormancy/auxin associated family protein	hormone metabolism.auxin.induced-regulated-responsive-activated
AT5G61380	8.42E-08	3.99E-05	ABI3-interacting protein 1 (AIP1)	hormone metabolism.abscisic acid.signal transduction; signalling.receptor kinases
AT3G62550	5.92E-08	3.37E-05	universal stress protein	hormone metabolism.ethylene.induced-regulated-responsive-activated
AT2G39900	4.89E-08	3.18E-05	LIM domain-containing protein	development.unspecified
AT2G16700	8.23E-07	0.0001225	actin depolymerizing factor 5 (ADF5)	cell.organisation
AT3G05220	2.42E-07	6.71E-05	heavy-metal-associated domain-containing protein	not assigned.no ontology
AT5G62720	3.10E-06	0.0002776	integral membrane HPP family protein	not assigned.no ontology
AT2G15890	3.47E-07	8.22E-05	expressed protein	not assigned.unknown
AT1G74440	2.81E-06	0.0002686	expressed protein	not assigned.no ontology
AT3G04550	3.43E-07	8.22E-05	expressed protein	not assigned.unknown
AT1G16840	1.98E-06	0.0002134	expressed protein	not assigned.unknown
AT4G02370	3.57E-06	0.0003055	expressed protein	not assigned.unknown
AT4G32340	1.38E-07	4.62E-05	expressed protein	not assigned.unknown

AT3G47160	1.76E-07	5.48E-05	expressed protein	not assigned.no ontology
AT3G61100	6.64E-07	0.0001111	expressed protein	not assigned.unknown
AT5G54970	5.70E-07	0.0001053	expressed protein	not assigned.unknown

Cluster 7

Locus ID	p-value	q-value	Annotation	MapMan Category
AT2G37680	4.23E-07	9.16E-05	phytochrome A specific signal transduction component	signalling.light
AT3G20810	2.33E-08	2.34E-05	transcription factor jumonji (jmjC) domain-containing protein	RNA.regulation of transcription.JUMONJI family
AT3G48360	1.58E-07	5.13E-05	speckle-type POZ protein	RNA.regulation of transcription.Transcriptional Adaptor Zinc Bundle (TAZ) domain family
AT3G60530	1.79E-06	0.0002046	zinc finger (GATA type) family protein	RNA.regulation of transcription.C2C2(Zn) GATA transcription factor family
AT4G05330	5.84E-07	0.0001053	zinc finger and C2 domain protein	RNA.regulation of transcription.C2H2 zinc finger family
AT4G36970	4.99E-07	9.78E-05	remorin family protein	RNA.regulation of transcription.unclassified
AT5G03470	1.87E-06	0.0002095	serine/threonine protein phosphatase 2A	protein.postranslational modification
AT3G59310	1.62E-06	0.0001919	expressed protein	protein.targeting
AT2G38290	2.92E-06	0.0002741	ammonium transporter 2	transport.ammonium
AT2G02390	2.87E-06	0.0002718	glutathione S-transferase zeta 1	misc.glutathione S transferases
AT1G47210	3.63E-06	0.0003058	cyclin family protein	cell.cycle
AT1G28330	5.88E-07	0.0001053	dormancy-associated protein	development.unspecified
AT4G21450	2.24E-07	6.37E-05	vesicle-associated membrane family protein	not assigned.no ontology
AT3G49780	5.34E-07	0.0001021	phytosulfokines 3	not assigned.no ontology
AT5G10860	6.61E-07	0.0001111	CBS domain-containing protein	not assigned.no ontology
AT3G18920	3.85E-07	8.76E-05	unknown	
AT5G42900	2.24E-08	2.34E-05	expressed protein	not assigned.unknown
AT4G30500	3.63E-06	0.0003058	expressed protein	not assigned.unknown
AT2G37480	1.93E-06	0.0002131	expressed protein	not assigned.unknown
AT1G26665	3.44E-08	2.61E-05	expressed protein	not assigned.unknown
AT3G15630	5.10E-07	9.91E-05	expressed protein	not assigned.unknown
AT4G19390	9.34E-08	4.17E-05	expressed protein	not assigned.unknown
AT3G53670	1.38E-07	4.62E-05	expressed protein	not assigned.unknown
AT5G11070	1.32E-06	0.0001668	expressed protein	not assigned.unknown

Supplementary Table S2. Genes involved in starch metabolism.

Gene	Locus	Annotation	Publications
GBSS	At1g32900	starch synthase, AtGBSS1	[1]
SS1	At5g24300	starch synthase, AtSS1	[2]
SS2	At3g01180	starch synthase, AtSS2	[3]
SS3	At1g11720	starch synthase, AtSS3	[4]
SS4	At4g18240	starch synthase, AtSS4	[5]
SS6	At5g65685	starch synthase, AtSS6	[6]
BE1	At3g20440	starch branching enzyme, AtBE1	[7]
BE2	At5g03650	starch branching enzyme, AtBE2, sbe2.2	[8]
BE3	At2g36390	starch branching enzyme, AtBE3, sbe2.1	[9]
ISA1	At2g39930	isoamylase-type debranching enzyme, AtISA1, AtISO1, ISO1	[10]
ISA2	At1g03310	isoamylase-type debranching enzyme, AtISA2, AtISO2, ISO2	[9]
ISA3	At4g09020	isoamylase-type debranching enzyme, AtISA3, AtISO3, ISO3	[11]
PU1	At5g04360	pullulanase-type starch debranching enzyme, limit dextrinase	[12]
DPE1	At5g64680	disproportionating enzyme, AtDE1, DPE1	[13]
DPE2	At2g40840	disproportionating enzyme, DPE2, AtDE2	[14]
BAM1	At4g15210	beta amylase, AtBAM1, ATb-Amy, RAM1	[15]
BAM2	At2g32290	beta amylase, AtBAM2	[15]
BAM3	At3g23920	beta amylase, AtBAM3	[15]
BAM4	At2g45880	beta amylase, AtBAM4	[16]
BAM5	At5g45300	beta amylase, AtBAM5	[16]
BAM6	At5g55700	beta amylase, AtBAM6	[17]
BAM7	At4g00490	beta amylase, AtBAM7	[17]
BAM8	At4g17090	beta amylase, ct-BMY, AtBAM8	[18]
BAM9	At5g18670	beta amylase, AtBAM9	[17]
AAM1	At1g76130	alpha amylase, AtAAM1	[19]
AAM2	At4g25000	alpha amylase, AtAAM2	[20]
AAM3	At1g69830	alpha amylase, AtAAM3	[20]
PHS1	At3g29320	phosphorylase, starch phosophorylase, AtPHS1, PHS	[21]
PHS2	At3g46970	phosphorylase, starch phosophorylase, AtPHS2, PHS	[22]
GWD1	At1g10760	Alpha-glucan water dikinase, GWD, R1, SEX1 At1g10760	[23]
PGI1	At4g24620	Phosphoglucoisomerase	[24]
PGM1	At5g51820	Phosphoglucomutase, PGM1	[25]
APS1	At5g48300	ADP-glucose pyrophosphorylase small subunit 1, AGP, ADG1	[26]
APL1	At5g19220	ADP-glucose pyrophosphorylase large subunit 1, AGP, ADG2	[27]
APL2	At1g27680	ADP-glucose pyrophosphorylase large subunit 2, AGP	[28]
APL3	At4g39210	ADP-glucose pyrophosphorylase large subunit 3, AGP	[29]
APL4	At2g21590	ADP-glucose pyrophosphorylase large subunit 4, AGP	[29]

Supplementary Table S3. Genes with the most significantly changes in transcript levels in the "Genotype" comparison between antisense-*ACLA* and WT over the SD diurnal cycle (FDR controlled at 0.2). Supplement to Figure 6A. Carbohydrate metabolic genes, genes involved in transcription.

Cluster G1

Locus ID	p-value	q-value	Annotation	MapMan Category
At4g14090	0.00011	0.1235	anthocyanidin 5-O-glucosyltransferase	misc.UDP glucosyl and glucoronyl transferases
At1g61810	3.51E-05	0.10362	glycosyl hydrolase family 1 protein	misc.gluco-, galacto- and mannosidases
At4g19840	0.00055	0.1891	lectin-related	misc.myrosinases-lectin-jacalin
At4g17030	0.00019	0.13796	expansin-related	cell wall.modification
At2g45400	6.49E-05	0.12212	dihydroflavonol 4-reductase family	secondary metabolism.flavonoids.flavonols
At2g03750	9.21E-05	0.1235	sulfotransferase family protein	lipid metabolism.'exotics' (steroids, squalene etc)
At2g37870	1.39E-06	0.03134	protease inhibitor/seed storage/lipid transfer protein (LTP)	misc.protease inhibitor/seed storage/lipid transfer protein (LTP) family protein
At2g28190	0.00011	0.1235	superoxide dismutase	redox.dismutases and catalases
At1g68570	0.00055	0.1891	proton-dependent oligopeptide transport	transport.peptides and oligopeptides
At1g17350	0.0006	0.19786	auxin-induced protein	hormone metabolism.auxin.induced-regulated-responsive-activated
At4g37580	3.69E-05	0.10362	N-acetyltransferase	hormone metabolism.ethylene.synthesis-degradation
At4g15910	0.00014	0.13796	drought-responsive protei	stress.abiotic.drought/salt
At4g23670	0.00051	0.1891	major latex protein-related	stress.abiotic.unspecified
At2g06190	0.0003	0.15473	gypsy-like retrotransposon family	DNA.synthesis/chromatin structure
At4g37510	0.00019	0.13796	ribonuclease III family protein	RNA.processing
At3g25710	0.00049	0.18888	basic helix-loop-helix (bHLH) family protein	RNA.regulation of transcription.bHLH,Basic Helix-Loop-Helix family
At1g75240	0.00029	0.15376	zinc finger homeobox family protein	RNA.regulation of transcription.unclassified
At3g06160	1.27E-05	0.07158	transcriptional factor B3 family protein	RNA.regulation of transcription.B3 transcription factor family
At5g35330	0.0002	0.13796	methyl-CpG-binding domain-containing protein	RNA.regulation of transcription.Methyl binding domain proteins
At1g76010	0.0004	0.17154	expressed protein	RNA.regulation of transcription.putative DNA-binding protein
At3g16560	0.00039	0.17154	protein phosphatase 2C-relate	protein.postranslational modification
At4g07670	0.00021	0.13796	protease-associated (PA) domain-containing protein	protein.degradation
At5g64150	0.00041	0.17586	methylase family protein	not assigned.no ontology
At2g40490	9.30E-06	0.07158	uroporphyrinogen decarboxylase	tetrapyrrole synthesis
At3g50440	0.00023	0.13796	similar to esterase	not assigned.no ontology
At2g46640	0.00036	0.17154	expressed protein	not assigned.unknown
At1g63420	0.00039	0.17154	expressed protein	not assigned.unknown
At3g59670	0.00025	0.14249	expressed protein	not assigned.unknown
At5g02550	0.0005	0.18888	expressed protein	not assigned.unknown

Cluster G2

Locus ID	p-value	q-value	Annotation	MapMan Category
At4g34135	0.00018	0.13796	UDP-glucoronosyl/UDP-glucosyl transferase	misc.UDP glucosyl and glucoronyl transferases
At1g59960	4.76E-05	0.10362	aldo/keto reductase	minor CHO metabolism.others
At1g35230	0.00044	0.18107	arabinogalactan-protein	cell wall.cell wall proteins.AGPs
At5g05600	0.00017	0.13796	flavonol synthase	secondary metabolism.flavonoids.anthocyanins
At5g35735	0.00038	0.17154	auxin-responsive family protein	hormone metabolism.auxin.induced-regulated-responsive-activated

At5g20810	0.00047	0.18543	auxin-responsive protein	hormone metabolism.auxin.induced-regulated-responsive-activated
At1g75030	0.00024	0.13982	pathogenesis-related thaumatin family protein	stress.biotic
At3g06050	0.00055	0.1891	redox homeostasis	stress.abiotic.unspecified; redox.periredoxins
At3g25010	0.00055	0.1891	disease resistance family protein	stress.biotic
At2g26020	0.00021	0.13796	plant defensin-fusion protein	metal handling.binding, chelation and storage; stress.biotic
At5g44420	0.00011	0.1235	plant defensin protein	metal handling.binding, chelation and storage; stress.biotic
At4g04150	0.00017	0.13796	gypsy-like retrotransposon family	DNA.synthesis/chromatin structure.retrotransposon/transposase.gypsy-like retrotransposon
At1g80840	0.00028	0.15376	WRKY family transcription factor	RNA.regulation of transcription.WRKY domain transcription factor family
At3g57230	0.00023	0.13796	Root Expressed; MADS-box protein	RNA.regulation of transcription.MADS box transcription factor family
At5g03720	9.69E-05	0.1235	member of Heat Stress Transcription Facto	stress.abiotic.heat; RNA.regulation of transcription.HSF,Heat-shock transcription factor family
At1g25220	0.00036	0.17154	Catalyzes the first step of tryptophan biosynthesis	amino acid metabolism.synthesis.aromatic aa.tryptophan
At1g76360	0.0001	0.1235	protein kinase	protein.postranslational modification
At5g52120	0.00046	0.18369	F-box family protein	protein.degradation.ubiquitin.E3.SCF.FBOX; cell.organisation
At3g01920	0.0001	0.1235	yrdC family protein	not assigned.no ontology
At3g25070	0.00016	0.13796	RPM1-interacting protein 4	not assigned.no ontology
At2g29460	1.63E-05	0.07366	glutathione S-transferase	misc.glutathione S transferases
At1g15140	8.50E-05	0.1235	oxidoreductase NAD-binding domain-containing protein	not assigned.no ontology
At5g52760	0.00011	0.1235	heavy-metal-associated domain-containing protein	not assigned.no ontology
At5g52750	0.0005	0.18888	heavy-metal-associated domain-containing protein	not assigned.no ontology
At1g65400	0.0003	0.15473	unknown	
At4g07380	5.05E-05	0.10362	expressed protein	not assigned.unknown
At4g29780	0.00018	0.13796	expressed protein	not assigned.unknown
At5g08240	0.00059	0.19743	expressed protein	not assigned.unknown

Cluster G3

**Locus ID**	**p-value**	**q-value**	**Annotation**	**MapMan Category**
At3g29810	0.00037	0.17154	phytochelatin synthetase family protein	cell wall.cellulose synthesis
At4g38880	3.05E-05	0.10362	amidophosphoribosyltransferase	nucleotide metabolism.synthesis.purine
At3g19950	0.00057	0.19297	zinc finger (C3HC4-type RING finger) family protein	RNA.regulation of transcription.unclassified
At1g72130	0.0002	0.13796	proton-dependent oligopeptide transport	transport.peptides and oligopeptides
At5g62730	0.0002	0.13796	proton-dependent oligopeptide transport	transport.peptides and oligopeptides
At3g26320	0.00043	0.18045	cytochrome P450 71B36	misc.cytochrome P450
At5g67310	4.33E-05	0.10362	cytochrome P450 family protein	misc.cytochrome P450
At3g17920	0.00034	0.17099	leucine-rich repeat family protein	not assigned.no ontology
At1g26360	0.00023	0.13796	a six-member gene family	not assigned.no ontology
At1g67330	0.00021	0.13796	expressed protein	not assigned.unknown
At1g13970	1.20E-05	0.07158	expressed protein	not assigned.unknown
At5g54300	0.00054	0.1891	expressed protein	not assigned.unknown

Supplementary Table S4. Genes with the most significantly changes in transcript levels in the "Genotype*Time" comparison between antisense-*ACLA* and WT over the SD diurnal cycle (FDR controlled at 0.45). Supplement to Figure 6B. Carbohydrate metabolic genes, genes involved in transcription.

Cluster G*T1

Locus ID	p-value	q-value	Annotation	MapMan Category
At1g26480	0.0011579	0.4190126	14-3-3 protein GF14 iota (GRF12)	signalling.14-3-3 proteins
At2g15480	0.0006609	0.4190126	UDP-glucoronosyl/UDP-glucosyl transferase family protein	misc.UDP glucosyl and glucoronyl transferases
At3g54920	0.0014628	0.4190126	pectate lyase	cell wall.degradation.pectate lyases and polygalacturonases
At4g20330	0.0015287	0.4190126	transcription initiation factor-related	RNA.processing
At2g18030	0.0002338	0.306494	peptide methionine sulfoxide reductase family protein	protein.aa activation
At1g65670	0.0015158	0.4190126	cytochrome P450 family protein	misc.cytochrome P450
At3g59670	0.0018365	0.4190126	expressed protein	not assigned.unknown
At5g10210	0.0002368	0.306494	expressed protein	not assigned.unknown
At5g55710	0.001277	0.4190126	expressed protein	not assigned.unknown
At4g07380	3.02E-05	0.1238788	expressed protein	not assigned.unknown
At1g05430	0.0008976	0.4190126	expressed protein	not assigned.unknown
At2g42130	0.001016	0.4190126	expressed protein	not assigned.unknown

Cluster G*T2

Locus ID	p-value	q-value	Annotation	MapMan Category
At2g44480	0.0008899	0.4190126	glycosyl hydrolase family 1	misc.gluco-, galacto- and mannosidases
At4g01890	0.0011546	0.4190126	glycoside hydrolase family 28 protein	cell wall.degradation.pectate lyases and polygalacturonases
At1g61290	0.0013614	0.4190126	syntaxin	cell. vesicle transport
At1g11040	0.0017662	0.4190126	DNAJ chaperone C-terminal domain-containing protein	stress.abiotic.heat
At4g14640	0.0005617	0.4190126	calmodulin-8 (CAM8)	signalling.calcium
At3g24220	0.0016779	0.4190126	9-cis-epoxycarotenoid dioxygenase	hormone metabolism.abscisic acid.synthesis-degradation
At4g07590	0.000201	0.306494	gypsy-like retrotransposon family	DNA.synthesis/chromatin structure.retrotransposon/transposase.gypsy-like retrotransposon
At5g43800	0.001201	0.4190126	copia-like retrotransposon family	DNA.synthesis/chromatin structure.retrotransposon/transposase.copia-like retrotransposon
At5g05510	0.0006095	0.4190126	protein kinase-related	protein.postranslational modification
At5g37450	0.001845	0.4190126	leucine-rich repeat transmembrane protein kinase	protein.postranslational modification
At1g01600	0.0003247	0.3502616	cytochrome P450	misc.cytochrome P450
At3g62280	0.0013627	0.4190126	GDSL-motif lipase/hydrolase family protein	misc.GDSL-motif lipase
At4g01410	0.0021122	0.4482418	harpin-induced family protein	not assigned.no ontology
At1g33340	0.0015072	0.4190126	epsin N-terminal homology (ENTH) domain-containing protein	not assigned.no ontology.epsin N-terminal homology (ENTH) domain-containing protein
AtMg01050	0.0009043	0.4190126	hypothetical protein	not assigned.no ontology
At1g13970	3.83E-05	0.1238788	expressed protein	not assigned.unknown
At1g01690	0.0009619	0.4190126	expressed protein	not assigned.unknown
At5g54790	0.0018046	0.4190126	expressed protein	not assigned.unknown
At4g39190	0.0016996	0.4190126	expressed protein	not assigned.unknown

Cluster G*T3

Locus ID	p-value	q-value	Annotation	MapMan Category
At1g47960	0.001194	0.4190126	invertase/pectin methylesterase inhibitor family protein	cell wall.pectin*esterases.PME; misc.invertase/pectin methylesterase inhibitor family protein
At4g14090	0.0014615	0.4190126	anthocyanidin 5-O-glucosyltransferase	misc.UDP glucosyl and glucoronyl transferases
At1g61810	0.000441	0.3806333	glycosyl hydrolase family 1 protein	misc.gluco-, galacto- and mannosidases
At3g18080	0.0006415	0.4190126	glycosyl hydrolase family 1 protein	misc.gluco-, galacto- and mannosidases
At2g05260	0.002081	0.4482418	lipase class 3 family protein	lipid metabolism.lipid degradation.lipases
At3g06160	0.0013065	0.4190126	transcriptional factor B3 family protein	RNA.regulation of transcription.B3 transcription factor family
At2g40490	0.0007156	0.4190126	uroporphyrinogen decarboxylase	tetrapyrrole synthesis
At1g63150	2.90E-05	0.1238788	pentatricopeptide (PPR) repeat-containing protein	not assigned.no ontology.pentatricopeptide (PPR) repeat-containing protein
At4g02240	0.002028	0.4482418	unknown	
At1g53620	2.98E-05	0.1238788	hypothetical protein	not assigned.no ontology.glycine rich proteins
At2g02570	0.0013419	0.4190126	expressed protein	not assigned.unknown
At2g16990	0.0017912	0.4190126	expressed protein	not assigned.unknown
At4g11870	0.002081	0.4482418	expressed protein	not assigned.unknown

Cluster G*T4

Locus ID	p-value	q-value	Annotation	MapMan Category
At1g62980	0.001313	0.4190126	expansin	cell wall.modification
At5g08030	0.0004111	0.3806333	glycerophosphoryl diester phosphodiesterase family protein	lipid metabolism.lipid degradation.lysophospholipases
At1g72890	6.57E-05	0.1700352	disease resistance protein	stress.biotic
At4g37580	0.001811	0.4190126	N-acetyltransferase	hormone metabolism.ethylene.synthesis-degradation
At2g03150	0.0011579	0.4190126	ATP/GTP-binding protein	signalling.G-proteins
At3g31340	0.0001483	0.306494	gypsy-like retrotransposon family	DNA.synthesis/chromatin structure.retrotransposon/transposase.gypsy-like retrotransposon
At2g19640	0.0004262	0.3806333	SET domain-containing protein	RNA.regulation of transcription.SET-domain transcriptional regulator family
At1g76360	0.0006505	0.4190126	protein kinase	protein.postranslational modification
At3g18120	0.0015143	0.4190126	F-box family protein-related	protein.degradation.ubiquitin.E3.SCF.FBOX
At3g63120	0.0016919	0.4190126	cyclin family protein	cell.cycle
At5g13170	0.000775	0.4190126	nodulin MtN3 family protein	development.unspecified
At5g67310	0.0002954	0.3476497	cytochrome P450 family protein	misc.cytochrome P450
At4g21320	0.0015119	0.4190126	heat-stress-associated 32-kD protein	not assigned.unknown
At1g11710	0.0015843	0.4190126	pentatricopeptide (PPR) repeat-containing protein	not assigned.no ontology.pentatricopeptide (PPR) repeat-containing protein
AtMg00850	0.0002299	0.306494	hypothetical protein	not assigned.unknown
At5g15890	0.0008317	0.4190126	expressed protein	not assigned.unknown
At4g02170	0.0008664	0.4190126	expressed protein	not assigned.unknown

References

1. Myers, A.M.; Morell, M.K.; James, M.G.; Ball, S.G. Recent progress toward understanding biosynthesis of the amylopectin crystal. *Plant Physiol.* **2000**, *122*, 989-997.
2. Pfister, B.; Lu, K.J.; Eicke, S.; Feil, R.; Lunn, J.E.; Streb, S.; Zeeman, S.C. Genetic Evidence That Chain Length and Branch Point Distributions Are Linked Determinants of Starch Granule Formation in Arabidopsis. *Plant Physiol.* **2014**, *165*, 1457-1474.
3. Zhang, X.; Myers, A.M.; James, M.G. Mutations affecting starch synthase III in Arabidopsis alter leaf starch structure and increase the rate of starch synthesis. *Plant Physiol.* **2005**, *138*, 663-674.
4. Vandromme, C.; Spriet, C.; Putaux, J.L.; Dauvillee, D.; Courseaux, A.; D'Hulst, C.; Wattebled, F. Further insight into the involvement of PII1 in starch granule initiation in Arabidopsis leaf chloroplasts. *New Phytol.* **2023**, *239*, 132-145.
5. Bürgy, L.; Eicke, S.; Kopp, C.; Jenny, C.; Lu, K.J.; Escrig, S.; Meibom, A.; Zeeman, S.C. Coalescence and directed anisotropic growth of starch granule initials in subdomains of *Arabidopsis thaliana* chloroplasts. *Nature Commun.* **2021**, *12*, 1-10.
6. Abt, M.R.; Pfister, B.; Sharma, M.; Eicke, S.; Bürgy, L.; Neale, I.; Seung, D.; Zeeman, S.C. STARCH SYNTHASE5, a noncanonical starch synthase-like protein, promotes starch granule initiation in Arabidopsis. *Plant Cell* **2020**, *32*, 2543-2565.
7. Wang, X.; Xue, L.; Sun, J.; Zuo, J. The Arabidopsis BE1 gene, encoding a putative glycoside hydrolase localized in plastids, plays crucial roles during embryogenesis and carbohydrate metabolism. *J. Integr. Plant Biol.* **2010**, *52*, 273-288.
8. Bossi, F.; Cordoba, E.; Dupre, P.; Mendoza, M.S.; Roman, C.S.; Leon, P. The Arabidopsis ABA-INSENSITIVE (ABI) 4 factor acts as a central transcription activator of the expression of its own gene, and for the induction of ABI5 and SBE2.2 genes during sugar signaling. *Plant J.* **2009**, *59*, 359-374.
9. Boyer, L.; Roussel, X.; Courseaux, A.; Ndjindji, O.M.; Lancelon-Pin, C.; Putaux, J.L.; Tetlow, I.J.; Emes, M.J.; Pontoire, B.; C, D.H.; et al. Expression of Escherichia coli glycogen branching enzyme in an Arabidopsis mutant devoid of endogenous starch branching enzymes induces the synthesis of starch-like polyglucans. *Plant Cell Environ.* **2016**, *39*, 1432-1447.
10. Yan, H.; Zhang, W.; Wang, Y.; Jin, J.; Xu, H.; Fu, Y.; Shan, Z.; Wang, X.; Teng, X.; Li, X.; et al. Rice LIKE EARLY STARVATION1 cooperates with FLOURY ENDOSPERM6 to modulate starch biosynthesis and endosperm development. *Plant Cell* **2024**, *36*, 1892-1912.
11. Nagler, M.; Nukarinen, E.; Weckwerth, W.; Nagele, T. Integrative molecular profiling indicates a central role of transitory starch breakdown in establishing a stable C/N homeostasis during cold acclimation in two natural accessions of *Arabidopsis thaliana*. *BMC Plant Biol.* **2015**, *15*, 284.
12. Delatte, T.; Umhang, M.; Trevisan, M.; Eicke, S.; Thorneycroft, D.; Smith, S.M.; Zeeman, S.C. Evidence for distinct mechanisms of starch granule breakdown in plants. *J. Biol. Chem.* **2006**, *281*, 12050-12059.
13. Backstrom, S.; Elfving, N.; Nilsson, R.; Wingsle, G.; Bjorklund, S. Purification of a plant mediator from *Arabidopsis thaliana* identifies PFT1 as the Med25 subunit. *Mol. Cell* **2007**, *26*, 717-729.

14. Li, X.; Ahmad, A.M.; Zhong, Y.; Ding, L.; Blennow, A.; Fettke, J. Starch phosphorylation regulates starch granule morphological homogeneity in *Arabidopsis thaliana*. *Plant Physiol.* **2024**, *194*, 2600-2615.
15. Monroe, J.D.; Storm, A.R.; Badley, E.M.; Lehman, M.D.; Platt, S.M.; Saunders, L.K.; Schmitz, J.M.; Torres, C.E. beta-Amylase1 and beta-amylase3 are plastidic starch hydrolases in Arabidopsis that seem to be adapted for different thermal, pH, and stress conditions. *Plant Physiol.* **2014**, *166*, 1748-1763.
16. Soyk, S.; Simkova, K.; Zurcher, E.; Luginbuhl, L.; Brand, L.H.; Vaughan, C.K.; Wanke, D.; Zeeman, S.C. The Enzyme-Like domain of Arabidopsis nuclear beta-Amylases is critical for dna sequence recognition and transcriptional activation. *Plant Cell* **2014**, *26*, 1746-1763.
17. David, L.C.; Lee, S.K.; Bruderer, E.; Abt, M.R.; Fischer-Stettler, M.; Tschopp, M.A.; Solhaug, E.M.; Sanchez, K.; Zeeman, S.C. BETA-AMYLASE9 is a plastidial nonenzymatic regulator of leaf starch degradation. *Plant Physiol.* **2022**, *188*, 191-207.
18. Schreier, T.B.; Umhang, M.; Lee, S.K.; Lue, W.L.; Shen, Z.; Silver, D.; Graf, A.; Muller, A.; Eicke, S.; Stadler-Waibel, M.; et al. LIKE SEX4 1 acts as a beta-Amylase-Binding Scaffold on starch granules during starch degradation. *Plant Cell* **2019**, *31*, 2169-2186.
19. Krokida, A.; Delis, C.; Geisler, K.; Garagounis, C.; Tsikou, D.; Pena-Rodriguez, L.M.; Katsarou, D.; Field, B.; Osbourn, A.E.; Papadopoulou, K.K. A metabolic gene cluster in *Lotus japonicus* discloses novel enzyme functions and products in triterpene biosynthesis. *New Phytol.* **2013**, *200*, 675-690.
20. Doyle, E.A.; Lane, A.M.; Sides, J.M.; Mudgett, M.B.; Monroe, J.D. An alpha-amylase (At4g25000) in Arabidopsis leaves is secreted and induced by biotic and abiotic stress. *Plant Cell Environ.* **2007**, *30*, 388-398.
21. Mérida, A.; Fettke, J. Starch granule initiation in *Arabidopsis thaliana* chloroplasts. *Plant J.* **2021**, *107*, 688-697.
22. Schopper, S.; Muhlenbock, P.; Sorensson, C.; Hellborg, L.; Lenman, M.; Widell, S.; Fettke, J.; Andreasson, E. Arabidopsis cytosolic alpha-glycan phosphorylase, PHS2, is important during carbohydrate imbalanced conditions. *Plant Biol. (Stuttg)* **2015**, *17*, 74-80.
23. Singh, A.; Compart, J.; AL‐Rawi, S.A.; Mahto, H.; Ahmad, A.M.; Fettke, J. LIKE EARLY STARVATION 1 alters the glucan structures at the starch granule surface and thereby influences the action of both starch‐synthesizing and starch‐degrading enzymes. *Plant J.* **2022**, *111*, 819-835.
24. Liu, H.C.; Chen, H.C.; Huang, T.H.; Lue, W.L.; Chen, J.; Suen, D.F. Cytosolic phosphoglucose isomerase is essential for microsporogenesis and embryogenesis in Arabidopsis. *Plant Physiol.* **2023**, *191*, 177-198.
25. Flutsch, S.; Horrer, D.; Santelia, D. Starch biosynthesis in guard cells has features of both autotrophic and heterotrophic tissues. *Plant Physiol.* **2022**, *189*, 541-556.
26. Funfgeld, M.; Wang, W.; Ishihara, H.; Arrivault, S.; Feil, R.; Smith, A.M.; Stitt, M.; Lunn, J.E.; Niittyla, T. Sucrose synthases are not involved in starch synthesis in Arabidopsis leaves. *Nat. Plants* **2022**, *8*, 574-582.
27. Tsai, H.L.; Lue, W.L.; Lu, K.J.; Hsieh, M.H.; Wang, S.M.; Chen, J. Starch synthesis in Arabidopsis is achieved by spatial cotranscription of core starch metabolism genes. *Plant Physiol.* **2009**, *151*, 1582-1595.

28. Ventriglia, T.; Ballicora, M.A.; Crevillen, P.; Preiss, J.; Romero, J.M. Regulatory properties of potato-Arabidopsis hybrid ADP-glucose pyrophosphorylase. *Plant Cell Physiol.* **2007**, *48*, 875-880.
29. Ventriglia, T.; Kuhn, M.L.; Ruiz, M.T.; Ribeiro-Pedro, M.; Valverde, F.; Ballicora, M.A.; Preiss, J.; Romero, J.M. Two Arabidopsis ADP-glucose pyrophosphorylase large subunits (APL1 and APL2) are catalytic. *Plant Physiol.* **2008**, *148*, 65-76.
